# Quantitative interaction mapping reveals an extended UBX domain in ASPL that disrupts functional p97 hexamers

**DOI:** 10.1038/ncomms13047

**Published:** 2016-10-20

**Authors:** Anup Arumughan, Yvette Roske, Carolin Barth, Laura Lleras Forero, Kenny Bravo-Rodriguez, Alexandra Redel, Simona Kostova, Erik McShane, Robert Opitz, Katja Faelber, Kirstin Rau, Thorsten Mielke, Oliver Daumke, Matthias Selbach, Elsa Sanchez-Garcia, Oliver Rocks, Daniela Panáková, Udo Heinemann, Erich E. Wanker

**Affiliations:** 1 Max Delbrück Center for Molecular Medicine, Robert-Rössle-Straße 10, 13125 Berlin, Germany; 2 Max-Planck-Institute for Coal Research, Kaiser-Wilhelm-Platz 1, 45470 Mülheim an der Ruhr, Germany; 3 Max Planck Institute for Molecular Genetics, Ihnestraße 63-73, 14194 Berlin, Germany; 4 Institute for Chemistry and Biochemistry, Freie Universität Berlin, Takustraße 6, 14195 Berlin, Germany

## Abstract

Interaction mapping is a powerful strategy to elucidate the biological function of protein assemblies and their regulators. Here, we report the generation of a quantitative interaction network, directly linking 14 human proteins to the AAA+ ATPase p97, an essential hexameric protein with multiple cellular functions. We show that the high-affinity interacting protein ASPL efficiently promotes p97 hexamer disassembly, resulting in the formation of stable p97:ASPL heterotetramers. High-resolution structural and biochemical studies indicate that an extended UBX domain (eUBX) in ASPL is critical for p97 hexamer disassembly and facilitates the assembly of p97:ASPL heterotetramers. This spontaneous process is accompanied by a reorientation of the D2 ATPase domain in p97 and a loss of its activity. Finally, we demonstrate that overproduction of ASPL disrupts p97 hexamer function in ERAD and that engineered eUBX polypeptides can induce cell death, providing a rationale for developing anti-cancer polypeptide inhibitors that may target p97 activity.

## Abstract (web summary)

The AAA+ ATPase p97 is an essential hexameric protein with multiple protein interaction partners and cellular functions. Here, the authors use interaction mapping to examine partner proteins of this large complex, and assess the effects of these proteins on the disassembly of the p97 complex.

Most biological processes are controlled by dynamic protein–protein interactions (PPIs), which form molecular networks of enormous complexity^1^,^2^. Several methods for the identification and characterization of PPIs have been developed, and some have been applied in large-scale studies^3^,^4^,^5^,^6^. Among these, the yeast two-hybrid (Y2H) system has been particularly attractive for PPI screening because binary interactions can be systematically and rapidly tested in an automated process at relatively low cost^7^,^8^. Y2H PPI data, however, are qualitative and do not provide information on interactions strength^9^, which is important for a better understanding of the cellular organization of proteins and the regulation of complex biological systems like signal transduction or gene expression^10^.

Recently, luminescence-based PPI mapping technologies such as LUMIER with BACON^11^,^12^ or DULIP^13^ have been established, which provide quantitative information about the association of proteins. The distinction between weak and strong interactions allows now the generation of quantitative interaction networks. This is very useful for the prioritization of PPIs for focused functional validation and for the identification of proteins that might be able to disrupt protein complexes^14^. Through the generation of a comprehensive quantitative chaperone interaction network, for example, the architecture of cellular protein homoeostasis pathways was elucidated^15^.

The AAA+ ATPase p97, also known as VCP (valosin-containing protein) in mammals or Cdc48 in yeast, is a homohexameric ring-shaped molecular machine that together with partner proteins controls a plethora of essential cellular processes such as ubiquitin-dependent protein degradation^16^, maintenance of quality control^17^ or homotypic membrane fusion^18^. It comprises four domains: a flexible N-terminal domain (N), two AAA+ ATPase domains (D1 and D2) and an unstructured C-terminal tail^19^,^20^. p97 is an essential protein, with orthologues in archaea and all eukaryotes. It is also highly relevant to disease, as it is mutated in familial cases of inclusion body myopathy associated with Paget disease of the bone and frontotemporal dementia (IBMPFD) and in amyotrophic lateral sclerosis^21^,^22^. There is increasing evidence that p97 is critical for proliferation of cancer cells^23^. p97 hexamers, therefore, are a target for cancer therapies. Small molecules inhibiting p97 ATPase activity have recently been identified^24^.

A large number of p97 interaction partners have been identified with proteomics methods and biochemical assays^25^,^26^. This includes 13 ubiquitin regulatory X (UBX) domain-containing proteins that commonly interact with the conserved N domain^27^. This domain is positioned at the periphery of p97 hexamers and acts as an allosteric regulator of ATPase activity^28^. Partners binding to it with high affinity might control p97 function by influencing its ATPase activity^29^. Information about the binding strengths of most known p97 interacting proteins is, however, not available.

To identify high-affinity binders, we decided to generate a quantitative p97 interaction map, involving automated Y2H screening followed by systematic validation of identified interactions in a dual luminescence-based co-immunoprecipitation (DULIP) assay. We discovered 12 high-confidence p97 interaction partners, four of which had been reported previously, including the UBX domain-containing protein ASPL (alveolar soft part sarcoma locus), which showed the highest binding strength in DULIP assays. Biochemical and structural studies revealed that a C-terminal fragment of ASPL is necessary and sufficient for p97 binding. This fragment causes efficient p97 hexamer disassembly and spontaneous formation of a heterotetrameric p97:ASPL protein complex. Strikingly, overproduction of ASPL caused disruption of functional p97 hexamers in cell models and blockage of ERAD. Finally, we showed that engineered ASPL-derived polypeptides can potently inhibit cell growth, suggesting that such molecules may be suitable as anti-cancer treatments.

## Results

### Identification of p97 interacting proteins

**Figure 1:**
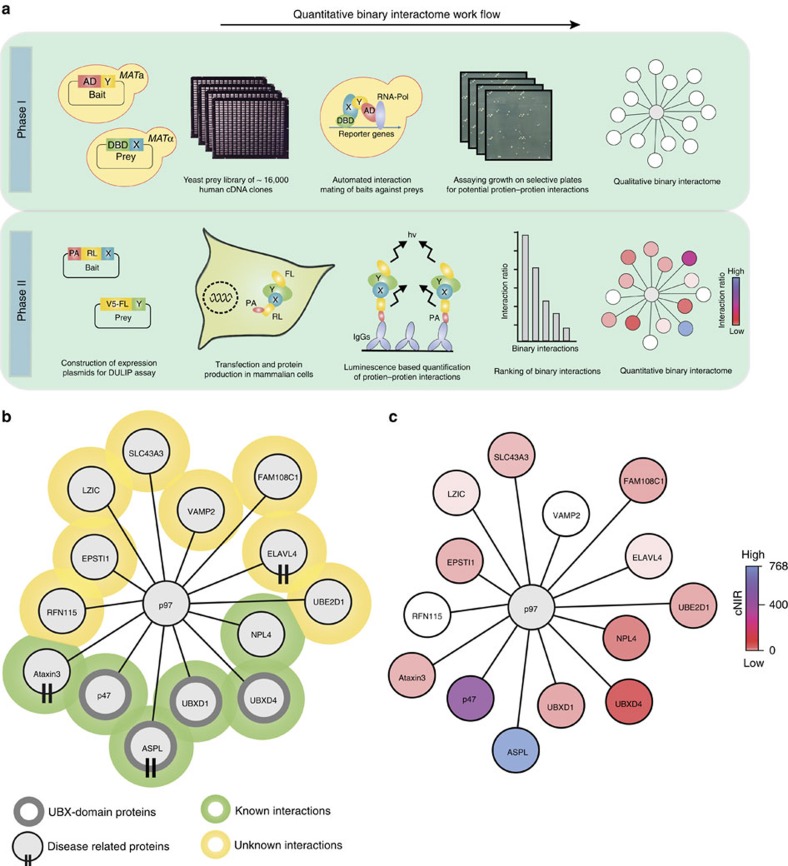
A quantitative p97 interaction network identifies ASPL as a strong binding partner of p97. (**a**) Schematic representation of the workflow for the generation of a quantitative binary p97 PPI interaction network using sequential applications of automated yeast two-hybrid technology (Phase I) and DULIP assays (Phase II). (**b**) Network graph of protein–protein interactions of p97 identified by automated Y2H assay. (**c**) Overlay of the DULIP assay data to the p97 Y2H network resulted in the generation of the quantitative binary interaction network for p97. In DULIP assays full-length p97 protein was utilized as bait.

We applied an automated yeast two-hybrid (Y2H) system^4^ to identify p97 interaction partners. Eight MATa yeast strains producing non-autoactivating p97 fragments (Supplementary Fig. 1a) were pooled and mated on YPD agar plates with ∼16,000 MATα yeast strains expressing human prey proteins. Interacting bait and prey proteins were finally identified through growth assays on selective agar plates. To increase our sampling the prey library was screened four times. Then, potential PPIs were tested pair wise in 4–6 independent mating experiments. Only pairs tested positive at least three times were considered as positive interactions. This interaction mating approach revealed 14 unique p97 partner proteins, 6 (∼43%) of which have been reported previously (Fig. 1a,b, and Supplementary Table 1).

We identified well-characterized p97 partner proteins such as p47, NPL4 or ATXN3 (ref. 16) and largely uncharacterized proteins such as UBXD4 (ref. 25). The majority of identified proteins interact with the N-terminus of p97 (Supplementary Table 1). This region contains the N domain, which has been shown to be critical for recruiting many adaptor proteins^30^. In our study, four UBX domain-containing proteins were identified that interact with N-terminal p97 fragments, confirming previously reported observations^16^. Overall, several p97 interactors with a functional role in ubiquitin-mediated protein degradation pathways were revealed (Supplementary Fig. 1b), supporting previous observations that p97 and its partners play a critical role in these processes^30^.

### DULIP-based ranking of p97 interacting proteins

To validate Y2H PPIs, a DULIP assay was applied, providing quantitative interaction information^13^. We systematically examined the interactions between protein-A-*Renilla* luciferase (PA-RL)-tagged fusion proteins and firefly luciferase (FL)-tagged fusion proteins in HEK293 cells. Through quantification of luciferase activities of precipitated protein complexes, background-corrected normalized interaction ratios (cNIRs) were calculated for all tested interactions (Fig. 1c and Supplementary Fig. 1c,d). These ratios are an indication of interaction strength, allowing the distinction of potentially high from low affinity interactions^13^. We used a stringent cNIR cutoff of >3 to define DULIP-positive PPIs^13^. Under these conditions 12 out of 14 (86%) tested Y2H interactions could be reproduced in DULIP assays with full-length p97 as a bait. The cNIRs varied over a range of ∼750-fold, suggesting that proteins interact with p97 with very different binding strengths. The cNIRs ranked the UBX domain-containing ASPL (Supplementary Fig. 1d) as the strongest p97 binder.

To substantiate this result, we finally employed biolayer interferometry (BLI)^31^ measurements to determine the binding affinity between purified His-tagged full-length ASPL and p97 (Supplementary Fig. 1e,f). The interaction between the full-length proteins was confirmed to be very strong with a dissociation constant (*K*_D_) of≈2 nM (Supplementary Fig. 1f), supporting our results with the DULIP assays (Fig. 1c and Supplementary Fig. 1d). Therefore, we selected the interaction between ASPL and p97 for more detailed characterization with biochemical and structural methods.

### ASPL binding to p97 involves an extended UBX domain

**Figure 2:**
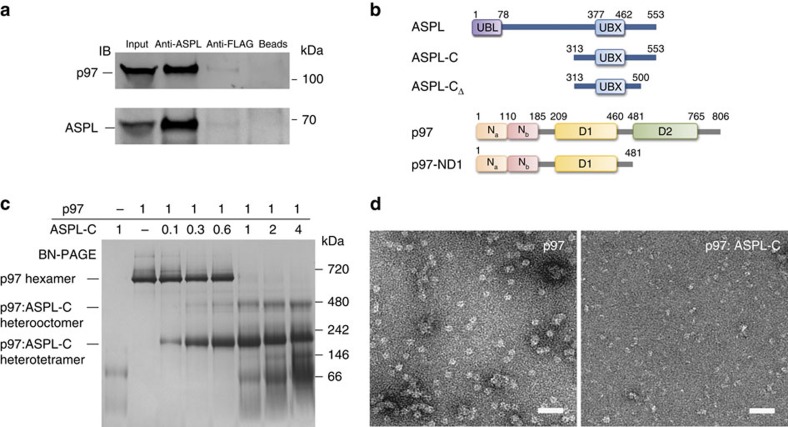
ASPL-C converts p97 hexamers into stable p97:ASPL-C heterooligomers. (**a**) Co-immunoprecipitation of a p97:ASPL protein complex from human brain homogenate using an anti-ASPL antibody. Anti-FLAG antibody and beads alone were used as negative controls. (**b**) Schematic representation of fragments and full-length ASPL and p97. Conserved protein domains are depicted: ubiquitin-like domain (UBL); ubiquitin regulatory-X domain (UBX); N-terminal protein binding domains (N_a_ and N_b_); ATPase domains (D1 and D2). (**c**) Blue-native gel stained with Coomassie Brilliant Blue, demonstrating the remodelling of p97 hexamers by ASPL-C in a concentration-dependent manner. p97 (10 μg) and ASPL-C (0.3, 0.6, 1.8, 3, 6 and 12 μg) were briefly mixed and incubated on ice for 5 min; then protein complexes were analysed by BN-PAGE. A 1:1 molar ratio of p97 monomers and ASPL-C was sufficient to promote the formation of p97:ASPL-C heterooligomers. (**d**) Negatively stained electron micrographs of purified p97 in the presence and absence of ASPL-C; scale bar 50 nm.

First, we assessed whether ASPL and p97 form a protein complex under physiological conditions. We found that p97 was efficiently co-immunoprecipitated by anti-ASPL antibodies from human post-mortem brain protein extract, but not by control antibodies or beads alone (Fig. 2a). This result was also confirmed when an anti-p97 antibody was utilized for co-immunoprecipitation (Supplementary Fig. 2a). Next, we defined the binding regions that are critical for the interaction. We first generated deletion fragments for ASPL and systematically investigated whether they associate with full-length p97 in DULIP assays (Supplementary Fig. 2b–c). As UBX domains in adaptor proteins have been shown to be critical for their interaction with p97 (ref. 32), we assumed this to be the case also for ASPL. Interestingly, cell-based PPI mapping revealed that the conserved UBX domain of ASPL (residues 377–462) alone does not interact with p97. However, an extended UBX (eUBX) domain-containing fragment (residues 313–553)—termed ASPL-C—showed strong binding to p97 (Fig. 2b and Supplementary Fig. 2c), indicating that amino acid residues located up- and downstream of the UBX domain are critical for the interaction with p97. This observation was independently verified by co-immunoprecipitations of V5-tagged truncated ASPL fragments and endogenous p97 from HEK293 cell extracts (Supplementary Fig. 3a).

Conversely, we also mapped the ASPL-binding region in p97 with DULIP assays (Supplementary Fig. 2d), revealing a relatively weak interaction between full-length ASPL and the N domain of p97 (aa 1–208). However, a stronger PPI was observed with the N-terminal p97-ND1 fragment (residues 1–480), which includes the N domain and the conserved D1 ATPase domain, indicating that both domains are critical for strong ASPL binding.

The interaction between ASPL-C and p97-ND1 was then examined with purified recombinant proteins using isothermal titration calorimetry (ITC), revealing a very stable association of these fragments *in vitro* (*K*_D_≈0.2 nM; Supplementary Fig. 3b).

Previous investigations demonstrated that binding of nucleotides such as ATP or ADP alters the conformation of p97 hexamers^33^, suggesting that they might also influence the interaction between ASPL and p97. GST pull-down assays showed that ATP, ADP or AMP-PNP do not significantly influence the binding of GST-ASPL-C to full-length p97 (Supplementary Fig. 3c), supporting the view that both proteins form a very stable complex *in vitro*.

### ASPL-C converts p97 hexamers into stable heterotetramers

We next examined the interaction between ASPL-C and p97 by blue native-PAGE (BN-PAGE), which allows the detection of protein complexes under non-denaturing conditions. We observed that p97 migrates at a molecular mass of ∼700 kDa (Fig. 2c), confirming the formation of stable hexamers^19^. However, these structures disappeared in the presence of increasing concentrations of ASPL-C while two lower molecular weight protein bands migrating at ∼230 and ∼480 kDa were detectable. This suggests that ASPL-C binding promotes the conversion of p97 hexamers into smaller p97:ASPL-C heterooligomers (Fig. 2c). Analysis by mass spectrometry revealed that these protein bands contain both p97 and ASPL-C polypeptides, supporting this hypothesis (Supplementary Fig. 4a,b). In strong contrast, heterooligomeric p97:ASPL-C protein complexes were not detectable when samples were analysed by SDS–polyacrylamide gel electrophoresis (SDS–PAGE) (Supplementary Fig. 3d).

Next, we analysed the interaction between p97 hexamers and ASPL-C by size-exclusion chromatography followed by static light scattering (SEC-SLS). A prominent peak was revealed at ∼230 kDa (Supplementary Fig. 4c), indicating the formation of stable p97:ASPL-C heterotetramers from p97 homohexamers.

Finally, we studied the effect of ASPL-C on p97 hexamers with negative-stain electron microscopy (EM). We observed typical ring-shaped hexameric p97 particles with diameters of ∼14 nm in the absence of ASPL-C (Fig. 2d), confirming previously published results^34^. These structures, however, were not detectable in the presence of ASPL-C, supporting the observations that ASPL-C binding promotes p97 hexamer disassembly (Fig. 2c and Supplementary Fig. 4c).

### Structure of p97-ND1:ASPL-C heterotetramers

**Figure 3:**
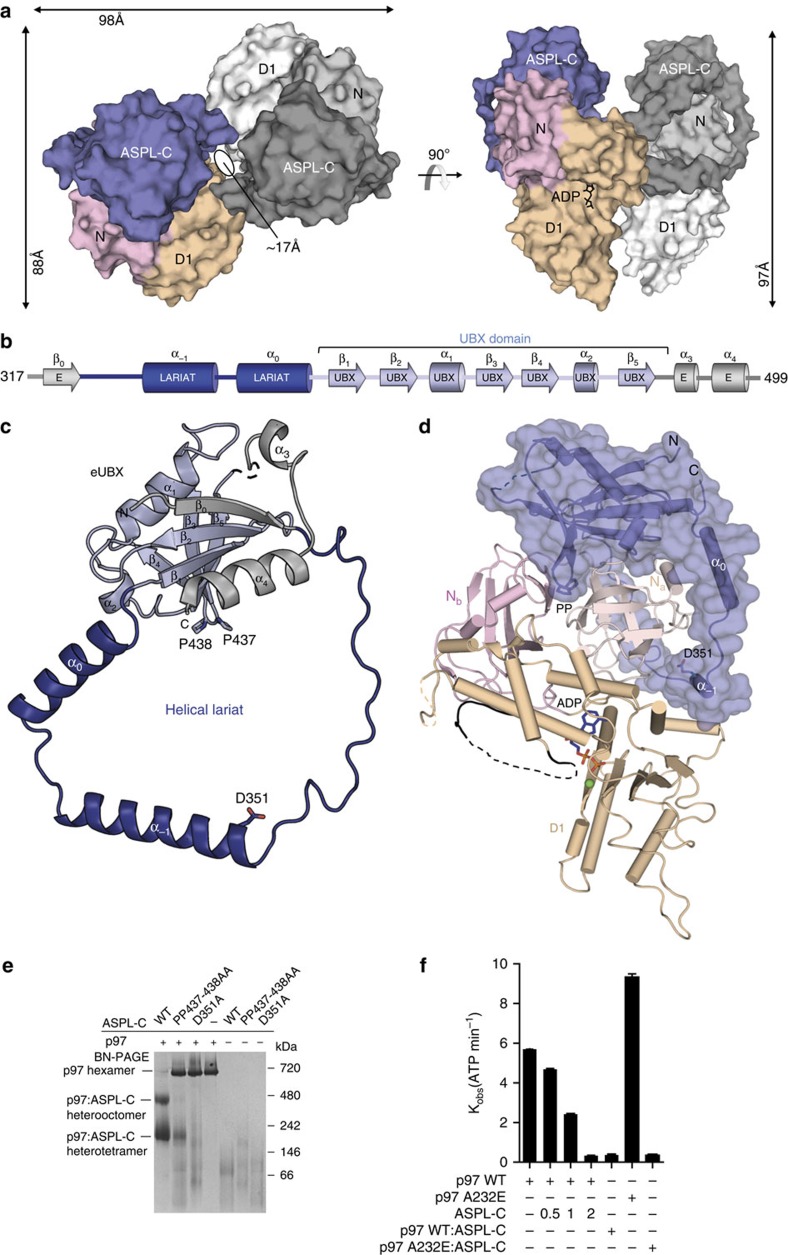
Structure of the ASPL-C:p97-ND1 heterotetramer. (**a**) Surface representation of a heterotetrameric p97-ND1:ASPL-C protein complex in two orthogonal orientations. One heterodimeric unit is displayed in shades of grey, the other is shown in multiple colours, blue (ASPL-C), pink (p97-N) and wheat (p97-D1). (**b**) Domain annotation and secondary structure of ASPL-C. The β-grasp fold (ββαββαβ) of the canonical UBX domain is coloured in light blue, the extensions β_0_, α_3_ and α_4_ in light grey, and the helical lariat in dark blue. (**c**) Ribbon-type representation of ASPL-C with colours as in **b**. (**d**) Structure of a single heterodimeric p97-ND1:ASPL-C unit observed within the heterotetramer. ASPL-C (blue) is shown as cartoon underneath a translucent molecular surface. The N_a_ (salmon colour) and N_b_ (pink) subdomains and the D1 domain (wheat colour) of p97 are displayed as cartoon structure. The partially disordered p97 D1-D2 linker is highlighted in black. ADP is depicted as stick model with a bound Mg^2+^ ion (green). (**e**) Coomassie-stained blue-native gel showing the effects of site-directed mutagenesis of conserved ASPL-C residues on the disassembly of p97 hexamers. ASPL-C (3 μg) or its indicated variants were combined with p97 (10 μg) and incubated on ice for 5 min. Then, samples were analysed by BN-PAGE. (**f**) ATPase activities of full-length p97 or its indicated variant in the presence or absence of ASPL-C were determined using a high-performance liquid chromatography-based assay. Molar ratios between ASPL-C and p97 are indicated. Wild-type (WT) p97 or the mutated protein p97-A232E were combined with a three-fold molar excess of ASPL-C and briefly incubated on ice. Then, the recombinant proteins were analysed by size-exclusion chromatography and heterotetrameric protein complexes (p97:ASPL-C or p97-A232E:ASPL-C) were isolated. ATPase activities of interacting recombinant proteins were analysed by high-performance liquid chromatography. Data are expressed as mean±s.e. (*n*=3).

To examine the molecular details of the interaction between p97 and ASPL, we determined the crystal structure of an ADP-bound p97-ND1 fragment in complex with ASPL-C at 2.46 Å resolution using molecular replacement (Supplementary Table 2). Two molecules each of p97-ND1 and ASPL-C were present in the asymmetric unit of the crystal, forming a compact heterotetrameric p97-ND1:ASPL-C protein complex (Fig. 3a). These results confirm the observations by BN-PAGE (Fig. 2c) that ASPL-C binding to p97 hexamers promotes the formation of stable heterooligomers (Fig. 2c and Supplementary Fig. 4c).

Assembly of the heterotetrameric p97-ND1:ASPL-C complex buries a total of 5,300 Å^2^ of solvent-accessible surface in both proteins. Two heterodimeric p97-ND1:ASPL-C units were found to be interlocked with each other, forming a central cavity with a diameter of ∼17 Å (Fig. 3a, Supplementary Movie 1). In each heterodimer, the N and D1 domains of p97-ND1 bind to ASPL-C primarily via polar interactions, burying an extensive interface area of 2,300 Å^2^. Two such heterodimeric units are assembled via a second interaction interface of 700 Å^2^ including many polar and van der Waals interactions. The overall structure of the p97-ND1 fragment in the heterotetramer shows high similarity to p97-ND1 in p47 bound hexamers (r.m.s.d.=0.95 Å; Supplementary Fig. 5a).

The conserved UBX-domain core (residues 390–466) in ASPL-C, which forms a β-grasp fold^27^, is also similar to previously reported structures of UBX domains in other p97-binding proteins such as p47 (r.m.s.d.=1.7 Å), FAF1 (r.m.s.d.=1.6 Å) and Npl4 (r.m.s.d.=2.2 Å) (Supplementary Fig. 5b). However, we found that the N- and C-terminal regions of the UBX domain in ASPL-C contain unique structural extensions, which were not observed in previously reported UBX-domain-containing proteins (Fig. 3b,c). We termed this region eUBX. It comprises the canonical UBX domain, three N-terminal extensions, β_0_, α_−1_, and α_0_ and two C-terminal extensions, α_3_ and α_4_, which make extensive contact with the β-grasp fold (Fig. 3c).

### ASPL-C binding to p97-ND1 involves an α-helical lariat

In the crystal structure, we observed that the association with p97-ND1 involves an α-helical lariat in ASPL-C (Fig. 3b–d). It consists of two long helices α_−1_ and α_0_ and a long loop which are inserted between β_0_ and β_1_ in the eUBX domain (Fig. 3b). Strikingly, the α-helical lariat wraps around almost the entire N_a_ subdomain of p97, suggesting that it is critical for the conversion of hexamers into heterotetramers (Fig. 3d). This assumption is supported by a superimposition of the structure of ASPL-C in p97-ND1 heterotetramers onto the structure of previously reported p97-ND1 homohexamers (Fig. 5c and Supplementary Fig. 5c). It shows that the α-helical lariat directly targets the D1:D1 interprotomer interface in p97 hexamers, a region critical for hexamer stability^35^, suggesting that it might play an important role in the dissociation of p97 homohexamers.

### Mutations in ASPL-C suppress p97 hexamer disassembly

To investigate whether point mutations in the UBX domain or the α-helical lariat influence ASPL-mediated p97 hexamer disassembly, we generated the mutants D351A and PP437-438AA and examined whether these ASPL-C variants can promote p97 hexamer dissociation. The conserved amino acid Asp351 was chosen for mutagenesis because it is located in the α-helical lariat (Supplementary Fig. 6a,b). Its exchange with Ala was expected to reduce the binding of the lariat to p97 hexamers. Pro437 and Pro438 locate in the canonical UBX domain of ASPL. They form a conserved *cis*-Pro touch-turn structure (Supplementary Fig. 6c,d), which docks to a hydrophobic pocket in p97, suggesting that it is critical for the association of ASPL-C with p97.

Next, we examined whether wild-type (wt) ASPL-C and its mutant variants D351A and PP437-438AA can promote p97 hexamer disassembly. Purified recombinant proteins were incubated with p97 hexamers; the spontaneous formation of putative heterooligomers was analysed by BN-PAGE and immunoblotting (IB). In contrast to wt ASPL-C, the mutants D351A and PP437-438AA showed a reduced ability to disassemble p97 hexamers *in vitro* (Fig. 3e and Supplementary Fig. 6e), indicating that both the α-helical lariat and the *cis*-Pro touch-turn structure in ASPL-C are critical for hexamer dissociation.

### ASPL binding inhibits p97 ATPase activity

To investigate whether ASPL-mediated remodelling of p97 hexamers influences ATP hydrolysis, we performed ATPase assays using purified recombinant proteins. In the absence of ASPL, p97 hexamers readily hydrolyzed ATP (Fig. 3f). When ASPL-C was added to p97 hexamers, however, this activity was reduced in a concentration-dependent manner, indicating that ASPL-mediated hexamer disassembly is associated with inhibition of ATP hydrolysis. A dramatically reduced ATPase activity was also observed when purified p97:ASPL-C heterotetramers were analysed (Fig. 3f).

Finally, we examined whether ASPL-C influences the ATPase activity of p97 hexamers harbouring the disease-causing mutation A232E (ref. 36). This mutation was previously shown to accelerate ATP turnover^28^, suggesting that hyperactive p97 hexamers are present in IBMPFD patients. In comparison to wt p97 hexamers, A232E hexamers exhibited an increased ATPase activity (Fig. 3f), which was significantly diminished in ASPL-C treated samples, supporting the results with wt p97.

### ASPL triggers a reorientation of the D2 ATPase domain in p97

**Figure 4:**
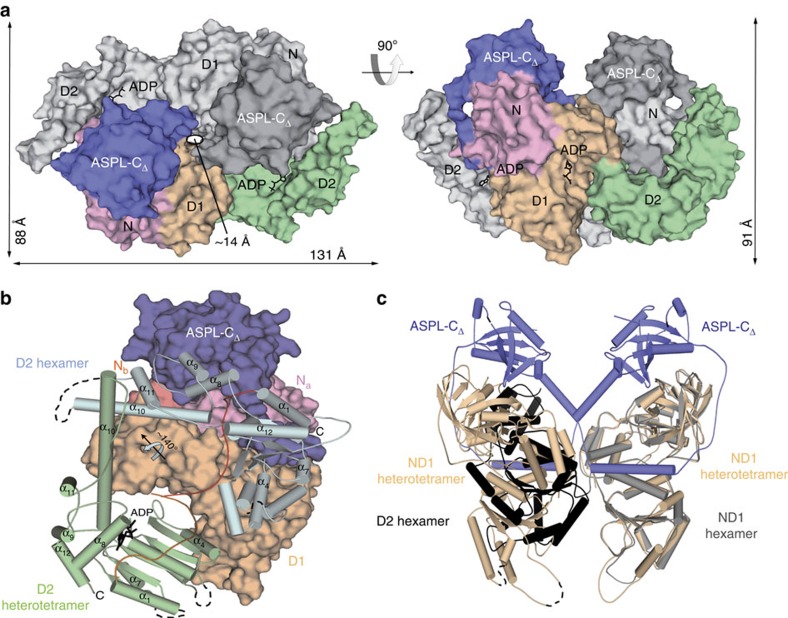
Remodelling of p97 hexamers by ASPL-C_Δ_ induces a rearrangement of the distal ATPase domain D2. (**a**) Surface representation of the heterotetrameric p97:ASPL-C_Δ_ protein complex in two orthogonal orientations. One heterodimeric unit is displayed in shades of grey, the other shows ASPL-C in blue, p97-N in pink, p97-D1 in wheat colour and p97-D2 in green. (**b**) Structural comparison of the orientation of the D2 domain observed in p97 hexamers and p97:ASPL-C_Δ_ heterotetramers. The N and D1 domains in the p97:ASPL-C_Δ_ heterodimeric unit are shown in surface representation in colours as in **a**, the D1-D2 linker (orange) and the D2 domain (green) are shown in cartoon representation. The relative orientation of the D2 domain in p97 hexamers is shown in light blue with the D1-D2 linker in red. (**c**) Structural comparison reveals the potential cause for the reorientation of the D2 domain in p97. Superposition of a single protomer taken from the p97 hexamer (N and D1 in grey, D2 in black) onto the p97-ND1:ASPL-C heterotetramer (p97-ND1 in wheat colour, ASPL-C in blue) demonstrates a steric interference between the D2 domain and a p97-ND1:ASPL-C heterodimeric unit.

Examination of the p97-ND1:ASPL-C heterotetramer structure (Fig. 3a) suggested that the formation of an analogous structure involving full-length p97 would be prevented by a steric clash between ASPL-C and the p97 D2 domain unless this domain adopts a structure that is different from the one observed in previously described p97 hexamers^19^. To address this question, we determined the crystal structure of nearly full-length p97 (residues 2–766) in complex with ASPL-C_Δ_ (residues 313–500) at a resolution of 3.4 Å (Fig. 4a and Supplementary Table 2). Protein complex formation was induced by an ASPL-C_Δ_ fragment that, compared with ASPL-C, lacks 53 disordered C-terminal amino acids (Fig. 2b). The asymmetric unit of the crystal contains one p97:ASPL-C_Δ_ heterodimeric unit (Supplementary Movie 2); p97:ASPL-C_Δ_ heterotetramers were generated by a crystallographic dyad axis (Fig. 4a).

We found that the overall structures of the p97:ASPL-C_Δ_ and p97-ND1:ASPL-C heterotetramers are very similar (r.m.s.d.=0.66 Å for the matching α-carbons), indicating that the N and D1 domains in p97 and the eUBX domain in ASPL have similar folds in both complexes. However, the p97:ASPL-C_Δ_ complex buries a larger surface of ∼7,000 Å^2^ (Supplementary Movie 3), suggesting that the D2 domain of p97 also contributes to the assembly of the heterotetramer (Fig. 4a). Overall, the structure of the D2 domain in p97:ASPL-C_Δ_ heterotetramers is very similar to the one previously described for p97 hexamers^19^, indicating that ASPL-C_Δ_-mediated remodelling of p97 hexamers does not alter the conformation of the D2 domain (Supplementary Fig. 7a). However, we found a different spatial orientation (Fig. 4b and Supplementary Fig. 7b–d), indicating that binding of ASPL-C_Δ_ to p97 triggers a drastic reorientation of the D2 domain. The movement of the D2 domain involves a 141° rotation around a hinge located at residue Leu464 in the D1-D2 linker region (Supplementary Movie 4), indicating that p97:ASPL-C_Δ_ heterotetramers (Fig. 4c) expose different surfaces than previously reported p97 hexamers.

Our structural data also provide an explanation for the reduced ATPase activity observed with p97:ASPL-C heterotetramers (Fig. 3f). Interprotomer contacts between D2 domains via arginine 635 are critical for efficient ATP hydrolysis at physiological temperatures^37^,^38^; in p97:ASPL-C_Δ_ heterotetramers, these contacts are not observed (Supplementary Fig. 7e).

### ASPL causes ERAD impairment and toxicity in mammalian cells

**Figure 5:**
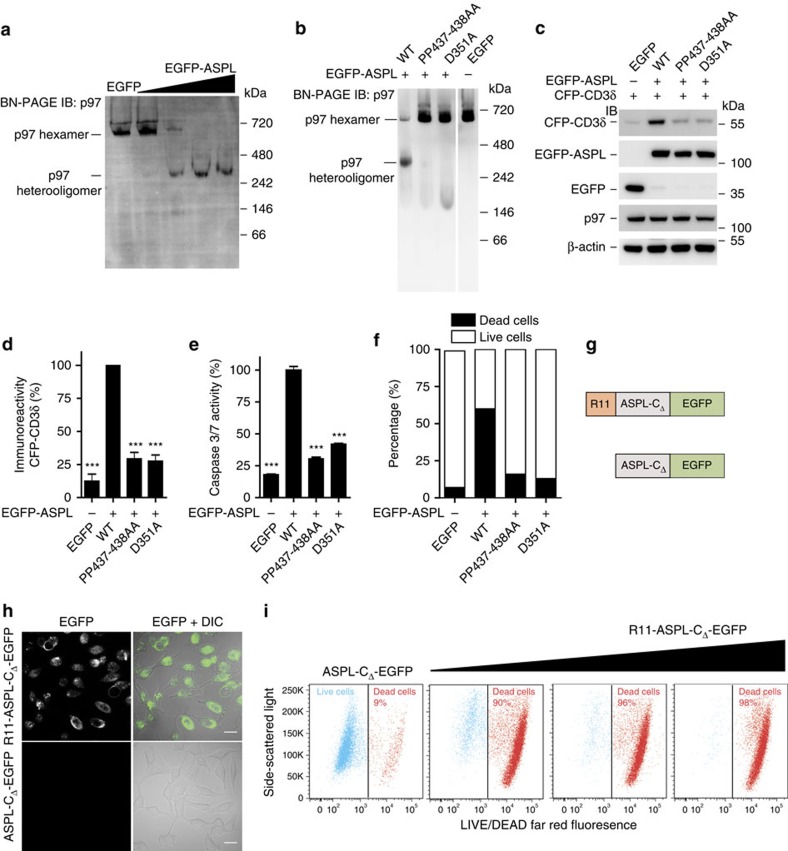
ASPL-induced disruption of p97 hexamers causes mammalian cell death. (**a**) Overproduction of EGFP-ASPL causes concentration-dependent disassembly of endogenous p97 hexamers in HEK293 cells. Protein extracts were analysed by BN-PAGE. p97-containing protein complexes were identified by IB using an anti-p97 antibody. (**b**) EGFP-ASPL and indicated variants of ASPL were overproduced in HEK293 cells. After 24 h, native protein complexes were resolved by BN-PAGE and immunoblotted to identify p97-containing protein complexes using an anti-p97 antibody. (**c**) CFP-CD3δ, EGFP-ASPL and indicated variants of ASPL were overproduced in HEK293 cells. After 24 h, total cell lysates were resolved by SDS–PAGE and immunoblotted to detect indicated proteins; antibodies: anti-CD3δ, anti-GFP, anti-p97 and anti-β-actin. (**d**) The levels of CFP-CD3δ in samples as indicated in **c** were quantified using an Aida image analyser. Data are expressed as mean±s.e. (*n*=3). ***P≤0.001 compared with WT-ASPL (Student's *t*-test). (**e**) EGFP-ASPL and indicated variants with point mutations were overproduced in HEK293 cells; activation of caspases 3/7 was monitored after 72 h. Data are expressed as mean±s.e.; (*n*=3). ***P≤0.001 compared with WT-ASPL (Student's *t*-test). (**f**) EGFP-ASPL and indicated variants with point mutations were overproduced in HEK293 cells for 72 h; cells were stained with LIVE/DEAD far-red fluorescence stain and live and dead cell populations were quantified using flow cytometry. 10,000 EGFP cells were analysed per each sample. (**g**) Schematic representation of R11- and EGFP-tagged ASPL-C_Δ_ recombinant proteins. (**h**) Confocal imaging micrographs of fixed HeLa cells showing the uptake of R11-ASPL-C_Δ_-EGFP protein into cells. Scale bars, 10 μm. (**i**) HeLa cells were treated for 24 h with the recombinant proteins R11-ASPL-C_Δ_-EGFP and ASPL-C_Δ_-EGFP (control protein), respectively; cells were stained with the LIVE/DEAD far-red fluorescence stain and live and dead cell populations were quantified using flow cytometry.

First, we examined whether overexpression of full-length ASPL can promote p97 hexamer disassembly in mammalian cells. We overproduced EGFP-tagged full-length ASPL in HEK293 cells and examined the formation of p97:ASPL-GFP protein complexes by BN-PAGE and IB. We found that EGFP-ASPL overproduction resulted in a concentration-dependent reduction of endogenous p97 hexamers (∼700 kDa) and promoted the formation of p97:ASPL-GFP heterooligomers (∼350 kDa) (Fig. 5a and Supplementary Fig. 8a,b). We further strengthened these findings by providing additional experimental evidence with size-exclusion chromatography studies. We confirmed that His-tagged, full-length ASPL can indeed disassemble preformed p97 hexamers and converts them into lower molecular weight protein complexes with a size of ∼300 kDa *in vitro* (Supplementary Fig. 8d,e).

Next, we assessed whether the ASPL mutants D351A and PP437-438AA promote p97 hexamer disassembly in cells. Analysis by BN-PAGE and IB revealed that the p97 hexamer-disassociation activity of the EGFP-tagged proteins D351A and PP437-438AA is significantly decreased in comparison with wt EGFP-ASPL (Fig. 5b and Supplementary Fig. 8c), supporting the results from the cell-free assays (Fig. 3e).

We then examined whether ASPL-mediated p97 hexamer disassembly influences described functions of p97 such as its role in endoplasmic reticulum-associated protein degradation (ERAD)^39^. We used the substrate CFP-CD3δ (ref. 40) to examine the effects of wt and mutant ASPL variants on p97 function in the ERAD pathway. The abundance of CFP-CD3δ was approximately fivefold increased in cells overproducing wt EGFP-ASPL compared with cells overproducing D351A or PP437-438AA (Fig. 5c,d), indicating that ASPL-mediated perturbation of p97 hexamer function is associated with ERAD inhibition in mammalian cells.

To substantiate this observation, we also assessed whether the endogenous levels of other known ERAD proteins are altered in response to ASPL overproduction. We overproduced the proteins EGFP-ASPL-WT, EGFP-ASPL-PP437-438AA or EGFP-ASPL-D351A in HeLa cells and quantified the abundance of newly synthesized L-azidohomoalanine (AHA)-labelled proteins^41^ using SILAC (stable isotope labelling with amino acids in cell culture^42^) -based quantitative mass spectrometry (Supplementary Fig. 9a). We found that overproduction of EGFP-ASPL-WT elevated the levels of the known ERAD pathway proteins BiP and Sec63 (Supplementary Fig. 9b), while such an effect was not observed with the mutated proteins EGFP-ASPL-PP437-438AA or EGFP-ASPL-D351A, substantiating the hypothesis that overproduction of wt ASPL perturbs ERAD function. A similar result was also obtained when the known p97 inhibitor NMS873 (ref. 43) was assessed with SILAC experiments in HeLa cells.

Perturbation of ERAD and p97 ATPase activity were previously shown to be associated with caspase activation in mammalian cells^44^. We therefore investigated in further studies whether overexpression of ASPL influences the activity of effector caspases in mammalian cells. We found that overproduction of wt ASPL in HEK293 cells resulted in an approximately fourfold increase in caspase-3/7 activity, while such an effect was not observed with the ASPL mutants D351A and PP437-438AA (Fig. 5e). This supports previous observations that ERAD impairment causes proteotoxic stress and activation of caspases^45^.

Finally, we employed a flow cytometry-based live–dead cell discrimination assay^46^ to examine whether EGFP-ASPL overproduction causes mammalian cell death. We observed a significant increase of dead cells when wt EGFP-ASPL was overproduced in HEK293 cells, while such an effect was not observed with the EGFP-tagged mutant proteins D351A and PP437-438AA (Fig. 5f). Similar results were also obtained when full-length wt ASPL or its truncated fragment ASPL-C_Δ_ were overproduced in HeLa, HCT116 or U2OS cancer cell lines (Supplementary Fig. 10a,b).

### Uptake of a short ASPL polypeptide decreases cell viability

To study whether ASPL-derived polypeptides influence cell viability, we generated purified, recombinant ASPL-C_Δ_-EGFP fusion proteins (wt ASPL-C_Δ_ and its mutated variants PP437-438AA and D351A) with and without an N-terminal poly-arginine (R11) tag (Fig. 5g and Supplementary Fig. 10c) and assessed whether they are taken up into mammalian cells when added to the culture medium. Analysis of cells by confocal microscopy showed that after 4 h of incubation the R11-tagged but not the untagged ASPL fusion proteins were detectable in HeLa cells (Fig. 5h and Supplementary Fig. 10d), indicating that the N-terminal poly-arginine tag promotes the uptake of proteins into cells. Next, we applied the flow cytometry-based LIVE/DEAD fluorescent cell-staining assay to investigate whether the uptake of the R11-ASPL-C_Δ_-EGFP fusion proteins into cells influences their viability. We observed a concentration-dependent increase of dead cells with the wt ASPL-C_Δ_ fusion protein but not with the mutant variants PP437-438AA and D351A (Fig. 5i and Supplementary Fig. 10e), confirming the results observed in overexpression experiments (Supplementary Fig. 10a,b).

## Discussion

In this study, we applied a two-step interaction mapping approach including automated Y2H screening and systematic validation of binary PPIs with a DULIP assay to identify high-affinity interaction partners for the AAA+ ATPase p97. p97 is a hexameric molecular machine that carries out diverse cellular functions in association with partner proteins^30^. Among others, p97 was shown to play a critical role in the degradation of misfolded proteins and to be involved in cellular processes such as Golgi membrane assembly^47^ or autophagosome formation^16^. Multiple partner and adaptor proteins of p97 have been identified with a variety of techniques^25^,^48^. However, detailed information on binding affinities or mechanisms is mostly missing. This information is of particular importance as previous investigations with p97 interactors indicate that they bind sequentially to specific domains in p97 (ref. 49), suggesting that high-affinity binders could displace other proteins from p97 hexamers and thereby influence their specific cellular functions.

Through our quantitative PPI mapping approach, we were able to rank the initially identified p97 interaction partners based on normalized interaction ratios (NIRs), an indicator of interaction strength^13^. This analysis revealed that among 14 proteins identified in Y2H assays, the UBX domain-containing protein ASPL binds most strongly to p97 (Fig. 1c). The interaction between ASPL and p97 has been reported previously^25^,^50^, information on interaction strength and whether the association with ASPL is stronger or weaker than other p97 interactions is unavailable, however. Evaluating our quantitative interaction data (Fig. 1c), we could group the identified p97 interaction partners according to their NIRs and define potential hierarchies among them that might be of relevance for a better understanding of p97 function.

Previous studies indicate that binding of ASPL or its orthologues to p97 promotes hexamer disassembly^51^,^52^,^53^, suggesting that the protein functions as a regulator of p97 ATPase activity. Our investigations of the interaction using biochemical and high-resolution structural methods support this view. Beyond previous findings, we could demonstrate that a C-terminal ASPL fragment harbouring an eUBX domain and an α-helical lariat are necessary and sufficient for the dissociation of p97 hexamers as well as the inhibition of ATPase activity (Fig. 3). Strikingly, we also found that ASPL-C-mediated hexamer disassembly causes the formation of stable p97:ASPL-C heterotetramers with a compact globular structure in cell-free assays.

**Figure 6:**
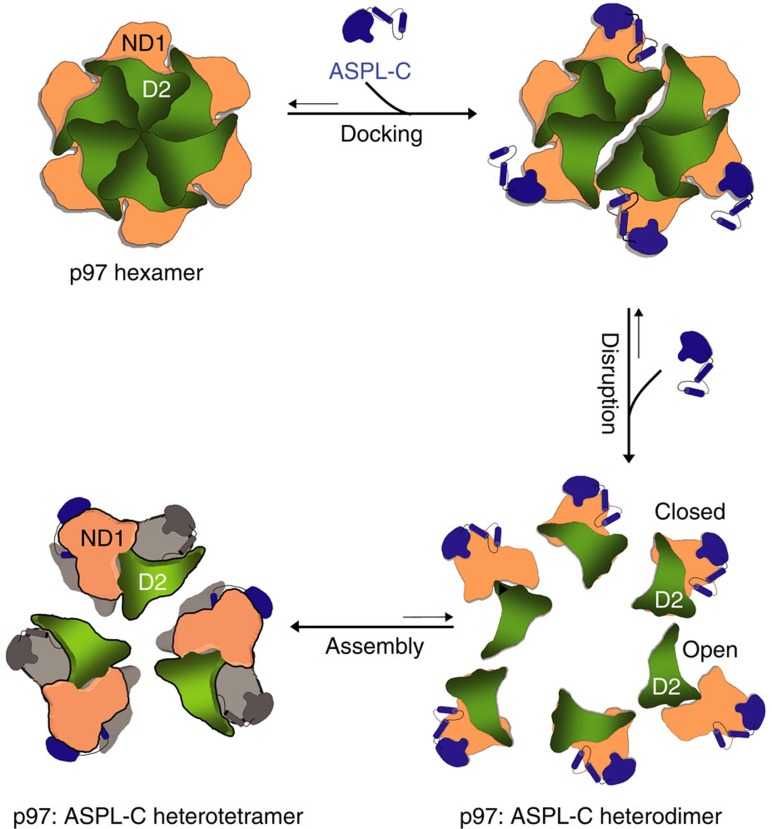
Structure-based model for the disassembly of p97 hexamers through the interaction with ASPL-C. ASPL-C-mediated disassembly of p97 hexamers is a multistep process. In the first step, ASPL-C docks onto the p97 surface via its *cis*-Pro touch-turn motif that is conserved in the eUBX domain. Next, the ASPL-C helical lariat disrupts the association of p97 protomers by targeting the D1 ring and binding to the p97 N_a_ subdomain. This results in the generation of six metastable p97:ASPL-C heterodimer units, which rapidly re-assemble into stable heterotetramers. Concomitantly, the D2 domain in p97 undergoes a ∼140° rotation from a closed to an open conformation, preventing the re-association of protomers into p97 hexamers.

This leads to the question of how the C-terminal fragments ASPL-C or ASPL-C_Δ_ (Fig. 2b) promote p97 hexamer disassembly. Our biochemical and structural studies suggest that the eUBX domain, which contacts the N domain in p97 through a conserved *cis*-Pro touch-turn motif, is important for the initial association with p97 hexamers, while the α-helical lariat structure is critical for hexamer dissociation (Fig. 3b,c and Supplementary Figs 5 and 6). Thus, both subdomains of ASPL-C or ASPL-C_Δ_ cooperate to target and disassemble p97 hexamers. While the importance of UBX domains in adaptor proteins for binding p97 hexamers is well documented^26^,^32^, an α-helical lariat structure that promotes hexamer disassembly has not been described previously. Our structural investigations suggest that the α-helical lariat in ASPL is a flexible structure that directly targets the D1:D1 interprotomer interface in p97 hexamers (Supplementary Fig. 5c), a region crucial for oligomer stability^35^. This interaction subsequently promotes the dissociation of p97 hexamers into ASPL-C-bound protomers, which spontaneously assemble into stable p97:ASPL-C heterotetramers. Interestingly, our structural studies revealed that the formation of p97:ASPL-C_Δ_ heterotetramers is accompanied by a reorientation of the D2 ATPase domain (Fig. 4). We suggest that this relocation of the D2 domain is critical for the assembly of stable p97:ASPL-C_Δ_ heterotetramers and the inhibition of ATPase activity (Fig. 3f). A mechanistic model outlining the potential steps of the ASPL-C-mediated p97 hexamer disassembly and the heterotetramer re-assembly process is shown in Fig. 6.

Based on these results, we primarily addressed the question of whether ASPL-derived fragments can be utilized as specific inhibitors to perturb p97 hexamer function in cell model systems. p97 is an essential protein and conserved across all eukaryotes. Furthermore, it was shown that p97 is involved in the development of various cancers^23^. A specific inhibitor of the protein would be a highly relevant tool to investigate its diverse functional roles and to evaluate its potential as a therapeutic target in human disease. We demonstrate that both overexpression of ASPL and the addition of a cell-permeable truncated ASPL fragment efficiently induces death of mammalian cells (Fig. 5f–i and Supplementary Fig. 10a,b), indicating that ASPL-derived polypeptides are potent inhibitors of p97 function. Overproduction of ASPL in cells was associated with inhibition of ERAD and mobilization of executioner caspases 3 and 7 (Fig. 5c–e), suggesting that high levels of ASPL induce cellular toxicity through inhibition of p97-dependent protein degradation pathways. Our investigations are in good agreement with studies of the small-molecule inhibitors of p97 such as DBeQ and CB-5083, which were shown to potently inhibit cancer cell growth, block ERAD and activate executioner caspases^43^,^44^,^54^,^55^,^56^. Our findings provide a rationale for targeting the N and D1 domains in p97, thereby disrupting the hexamer structure and inhibiting the ATPase activity of p97. Based on the high-resolution structures we describe, we envision that highly specific therapeutic molecules should be designed that directly target p97 hexamers and inhibit their function in cancer cells.

## Methods

### Production and purification of recombinant proteins

Full-length cDNAs encoding human ASPL and p97 were obtained from the Mammalian Gene Collection library. All plasmids were generated using standard PCR-based cloning strategies; Sanger sequencing verified nucleotide sequences. Human C-terminally His-tagged full-length ASPL (residues 1–553), ASPL-C (residues 313–553), ASPL-C_Δ_ (residues 313–500), R11-ASPL-C_Δ_-EGFP (and its variants: PP437-438AA, D351A), ASPL-C_Δ_-EGFP and ASPL-C variants containing the indicated mutations were produced from pQLinkG^57^ as N-terminally tagged GST fusions followed by a TEV (Tobacco Etch Virus) protease cleavage site in the *Escherichia coli* host strain BL21 DE3 Rosetta2. Bacteria were grown to an optical density of 1 at 37 °C; gene expression was induced using 1 mM isopropyl-β-D-thio-galactopyranoside (IPTG) and bacteria were grown overnight at 18 °C. Cells resuspended in PBS containing 5% glycerol, 1 μM Benzonase (Roche) and a complete, EDTA-free protease inhibitor cocktail (Merck) were disrupted by brief sonication (UW 2200, Bandelin). After removal of the cell debris by centrifugation, the supernatant was incubated with glutathione-Sepharose 4B beads (GE Healthcare). Subsequently, beads were washed with PBS, and the bound proteins were eluted with PBS containing 30 mM reduced glutathione (Sigma). Eluted proteins were incubated with His_6_-tagged TEV protease and dialyzed overnight against buffer A (20 mM HEPES/NaOH pH 7.4, 200 mM NaCl and 2 mM DTT). GST and TEV protease were separated from ASPL-C using glutathione-Sepharose beads and Ni-NTA resin (Qiagen) followed by size-exclusion chromatography on a Superdex S200 column (GE Healthcare) in buffer A. Purified ASPL-C fractions were pooled, concentrated (10,000 MWCO, Amicon) and flash-frozen in liquid nitrogen.

Human p97 (residues 2–806), nearly full-length p97 (residues 2–766) and p97-ND1 (residues 1–480) were produced as His_6_-tagged fusions from pQLinkH (ref. 57) in *E. coli* BL21 DE3 Rosetta2, using the conditions described for ASPL-C. The proteins were purified according to reference^19^. After affinity purification using Ni-NTA resin, the proteins were incubated with TEV protease and dialyzed against buffer A overnight, followed by size-exclusion chromatography on a Superdex S200 column (GE Healthcare) in buffer A. Purified p97 fractions were pooled, concentrated using ultrafiltration (30,000 molecular weight cutoff; Amicon) and flash-frozen in liquid nitrogen. Purified p97 protein was examined for the presence of bound nucleotides using reverse-phase chromatography (Hypersil ODS-2 C18 column). All biochemical experiments were performed with full-length p97 (residues 2–806).

### Crystallization and structure determination

Purified p97-ND1 (residues 1–480) was pre-incubated with 2 mM ADP and 5 mM MgCl_2_ for 1 h at 4 °C and mixed with purified ASPL-C (residues 313–553) in a 1:3 molar ratio. The reconstituted protein complexes were subjected to size-exclusion chromatography on a Superdex S200 column (GE Healthcare) in buffer A supplemented with 2 mM ADP and 5 mM MgCl_2_. The purified p97-ND1:ASPL-C protein complex was concentrated and supplemented with 15 mM KCl (300 nl at a concentration of 12.3 mg ml^−1^) and mixed with an equal volume of reservoir solution containing 23% PEG 3350, 0.3 M Li_2_SO_4_, 0.1 M HEPES/NaOH (pH 7.0). p97:ASPL-C_Δ_ protein complexes were prepared as described above for the p97-ND1:ASPL-C complex. The crystallization experiments were performed using the sitting-drop vapour-diffusion method at 4 °C with a Gryphon pipetting robot (Matrix Technologies Corporation) and a Rock Imager 1000 storage system (Formulatrix). Crystals of the p97:ASPL-C_Δ_ complex (300 nl at a concentration of 15.5 mg ml^−1^) were obtained by mixing an equal volume (200 nl) of reservoir solution (2 M ammonium sulfate, 0.1 M Bis-Tris pH 5.5) with the protein solution. Crystals of both complexes appeared after 2 weeks and were flash-frozen in liquid nitrogen in a cryo solution containing additionally 20% glycerol relative to the reservoir solution. All data were recorded at BL14.1 at BESSY II (Helmholtz-Zentrum Berlin, HZB) at a wavelength of 0.9184 Å, processed and scaled using the XDS suite^58^. Phases for the p97-ND1:ASPL-C complex were obtained by molecular replacement with Phaser^59^ using the p97-ND1 domain (PDB code 1S3S) as a search model. For p97:ASPL-C_Δ_, the fully refined p97-ND1:ASPL-C complex structure was used for molecular replacement. Both protein complex structures were manually built using COOT^60^ and iteratively refined using Phenix^61^ and Refmac^62^. The final p97-ND1:ASPL-C structure comprises amino acids 313–495 for ASPL-C and 21–480 for p97-ND1. Residues 313–316, 468–470 and 498–553 for ASPL-C and residues 1–20, 428–432 and 462–472 for p97-ND1 were disordered and therefore not visible in the electron density. The p97:ASPL-C_Δ_ complex consists of residues 317–499 for ASPL-C_Δ_ and 13–761 for p97. Unstructured regions were found for residues 313–316 in ASPL-C_Δ_ and residues 2–12, 427–433, 495–510, 585–596, 613–615 and 762–766 in p97. In the p97-ND1:ASPL-C complex, 99.8% of the residues were in the allowed regions of the Ramachandran map. In the p97:ASPL-C_Δ_ structure 100% of all residues were in the allowed regions of the Ramachandran map. The Ramachandran statistics were analysed by Molprobity^63^ for both complexes. Figures and domain superpositions were generated with PyMol (http://www.pymol.org). The Dali server^64^ was used to search for structural homologues of ASPL. The analysis of the p97:ASPL interface was performed with PISA^65^. The rearrangement of the p97 D2 domain was determined as a hinge-like 141° rotation centred at Leu^464^ coupled to a 3 Å translation along the rotation axis by DynDom^66^. The movies S1–4 were generated by using the program Pymol. The simulation of the D2 domain rearrangement was generated by using the morph server^67^.

### Co-immunoprecipitations

Human brain extracts were prepared from ∼4 g of brain and homogenized briefly in ice-cold PBS supplemented with 1% Triton X-100, complete protease inhibitor cocktail (Roche) and Benzonase (Merck). The homogenate was subjected to centrifugation at 20,000*g* for 10 min at 4 °C; the supernatant was collected and used for co-immunoprecipitation experiments as described previously with indicated modifications^68^. Magnetic beads coupled with protein G (Dynabeads; Invitrogen) were incubated with anti-ASPL antibody (Abnova, clone 3D10-1D11,1:500 dilution) for 1 h at 4 °C. Antibody bound to magnetic beads was crosslinked using 5 mM BS (ref. 3) (Thermo Scientific) according to the manufacturer's instructions. Magnetic beads with crosslinked antibody were incubated with human brain homogenate at 4 °C with rotation for 1 h. The bound protein complexes were washed briefly with PBS containing 1% Triton X-100, followed by elution of protein complexes with SDS sample buffer. Proteins were analysed by SDS–PAGE and western blotting using anti-ASPL (Abnova clone 3D10-1D11, 1:500 dilution) and anti-p97 (PROGEN, clone 58.13.3, 1:20,000 dilution) antibodies. Co-IP experiments in reverse direction were carried out using the Pierce Crosslink Immunoprecipitation Kit (cat. 26147) according to manufactures instructions using the anti-p97 antibody (LifeSpan Biosciences, cat. LS-C287469, 1:2,000 dilution).

Recombinant V5-tagged ASPL proteins were overproduced in human embryonic kidney cells 293 (HEK293). After 48 h media was aspirated and cells were washed briefly in PBS, lysed using a buffer containing 0.1% NP40, 20 mM HEPES/NaOH pH 7.4, 150 mM NaCl, 1.5 mM MgCl_2_, 1 mM EDTA and freshly supplemented with complete protease inhibitor cocktail (Roche), Benzonase nuclease (Merck). Lysis was carried out for 30 min on ice. The homogenate was briefly centrifuged on a table top centrifuge for 1–2 min to remove the cell debris and the clarified supernatant was incubated with V5-agarose beads (Abcam, cat. ab1229) for 1 h at 4 °C with rotation. The bound protein complexes were washed three times using a buffer containing 0.1% NP40, 20 mM HEPES/NaOH pH 7.4, 150 mM NaCl, 1.5 mM MgCl_2_ and 1 mM EDTA. This was followed by elution of the protein complexes with elution buffer containing primary amine pH 2.8 from Pierce Crosslink Immunoprecipitation Kit (cat. 26147). Proteins were analysed by SDS–PAGE and western blotting using anti-V5 (Abcam, cat. ab27671, 1:1000 dilution), anti-p97 (PROGEN, clone 58.13.3, 1:20,000 dilution) and β-actin (Sigma- Aldrich, cat. A5441, 1:40,000) antibodies.

### DULIP assay

Fusion proteins containing a protein A (PA)-renilla luciferase (RL) tag were co-produced together with V5-firefly luciferase (FL)-tagged proteins in HEK293 cells. After 48 h, cells were harvested, lysed in buffer B (0.1% NP40, 50 mM HEPES/NaOH pH 7.4, 150 mM NaCl, 1.5 mM MgCl_2_, 1 mM EDTA, 1 mM DTT) containing the Complete protease inhibitor cocktail (Roche) and Benzonase (Merck). Luciferase activity in protein extracts was measured to monitor the production of fusion proteins. Protein complexes were captured using IgG (Jackson Immunoresearch) immobilized to high-binding 96-well white plates (Greiner). Bound protein complexes were washed briefly with buffer B lacking NP40, and binding of the firefly-V5-tagged fusion protein (Co-IP) to the PA-Renilla-tagged fusion protein was quantified by measuring the firefly luciferase activity in a luminescence plate reader (TECAN Infinite M200). Renilla-luciferase activity was also measured as a control (IP). Luciferase activity was measured using the Dual-Glo Luciferase Assay System (Promega) following the manufacturer's instructions. Individual PPIs were tested as triplicates; experiments were repeated at least three times.

### Blue native-PAGE

Proteins were analysed by BN-PAGE according to the manufacturer's instructions (Invitrogen). Protein samples were resolved on 4–16% Bis-Tris BN-PAGE gels at 200 V at room temperature and visualized by Coomassie Brilliant Blue staining. The molar concentration of p97 was calculated in terms of its monomer for all the *in vitro* experiments (Supplementary Fig. 11). The intensity of the protein bands was quantified using the Aida image analyser software. Native p97-containing protein complexes from HEK293 cell extracts were resolved by BN-PAGE and analysed by western blotting using an anti-p97 antibody (PROGEN, clone 58.13.3, 1:20,000 dilution) (Supplementary Fig. 11). Western blotting of BN gels was performed according to the manufacturer's instructions.

### Negative-stain electron microscopy

An amount of 3.5 μl of purified p97 hexamers (residues 2–806) or p97:ASPL-C heterotetramers at a protein concentration of 10–20 ng μl^−1^ in buffer A (see Production and purification of recombinant proteins) were applied to freshly glow-discharged holey carbon grids (300 mesh R2/4 Quantifoil grids, Quantifoil Micro Tools GmbH, Jena, Germany) covered with an additional thin layer of continuous carbon and negatively stained with 2% (w/v) uranyl acetate. Samples were evaluated at 28,500-fold nominal magnification using a Philips CM100 electron microscope operated at 100 kV, which was equipped with a 1 k × 1 k F114 Fastscan CCD camera (TVIPS).

### ATPase assay

ATPase activities of 0.5 μM p97 in the presence and absence of ASPL-C were determined at 37 °C in ATPase buffer containing 20 mM HEPES/NaOH (pH 7.4), 40 mM NaCl, 10 mM MgCl_2_. Reactions were initiated by the addition of 250 μM ATP to samples. At different time points, reaction aliquots were taken, diluted 15-fold in ATPase buffer and quickly transferred to liquid nitrogen. Nucleotides in these samples were separated by a reversed-phase Hypersil ODS-2 C18 column (250 × 4 mm) with 10 mM tetrabutylammonium bromide, 100 mM potassium phosphate (pH 6.5), 7.5% acetonitrile as running buffer. Denatured proteins were adsorbed at a C18 guard column. Nucleotides were detected by absorption at 254 nm and quantified by integration of the corresponding peaks. Rates were derived from a linear fit to the initial reaction.

### Caspase 3/7 assays

HEK293 cells were grown at 37 °C and 5% CO_2_ in Dulbecco's modified Eagle medium (DMEM) supplemented with 1 g l^−1^ D-glucose, 10% fetal calf serum (FCS), penicillin (100 μg ml^−1^) and streptomycin (100 μg ml^−1^). Equal numbers of HEK293 cells were seeded on a 96-well microtiter plate. After 24 h, cells were transfected with pEGFP-C1 (Clontech) fusion constructs encoding ASPL or indicated ASPL variants using PEI (polyethyleneimine, Polysciences) for 72 h. The activation of caspases 3/7 was monitored using the Apo-ONE Homogeneous Caspase-3/7 Assay kit (Promega). The caspase-3/7 substrate added to each well was mixed briefly by shaking and incubated for 1 h at room temperature. The fluorescence signal was determined using a plate reader (TECAN Infinite M200). The expression of ASPL constructs and p97 was monitored by IB using anti-GFP (Abgent, cat. AM1009a,1:10,000 dilution) and anti-p97 (PROGEN, clone 58.13.3, 1:20,000 dilution) antibodies, respectively. The expression of β-actin was also monitored by IB using an anti-β actin antibody (Sigma-Aldrich, cat. A5441, 1:40,000).

### ERAD assay

CFP-tagged T-cell receptor subunit CD3δ, an ERAD model substrate, was co-expressed with EGFP-ASPL in HEK293 cells. After 24 h, cell lysates were prepared and the steady-state levels of CFP-CD3δ were analysed by SDS–PAGE and IB using an anti-CD3δ antibody (Santa Cruz, cat. SC-137137, 1:500) (Supplementary Fig. 11). The expression of ASPL constructs and p97 was monitored by IB using anti-GFP (Abgent, cat. AM1009a) and anti-p97 (PROGEN, clone 58.13.3, 1:20,000 dilution) antibodies, respectively. The expression of β-actin was also monitored by IB using an anti-β-actin antibody (Sigma-Aldrich, cat. A5441, 1:40,000).

### LIVE/DEAD assay

HEK293, HeLa (human cervical cancer cells), U20S (human osteosarcoma cells) and HCT116 (human colon carcinoma cells) cell lines were grown at 37 °C and 5% CO_2_ in DMEM supplemented with either 1 g l^−1^ D-glucose (HEK293, HeLa, U20S) or 4 g l^−1^ D-glucose (HCT116), 10% fetal calf serum, penicillin (100 μg ml^−1^) and streptomycin (100 μg ml^−1^). The LIVE/DEAD fixable far-red cell stain (Life Technologies) was used to discriminate live and dead cells according to the manufacturer's instructions. Briefly, 1 × 10^6^ cells were harvested, washed and treated with either the indicated ASPL-C_Δ_ recombinant proteins for 24 h or transfected with pEGFP-C1 (Clontech) fusion constructs encoding ASPL or indicated ASPL variants using PEI (polyethyleneimine, Polysciences) for 72 h. Cells were harvested and 1 μl of dye was added to each sample, mixed, and then incubated in the dark for 30 min at room temperature, washed and fixed in 2% formaldehyde in PBS for 15 min. Fixed cells were maintained in 300 μl PBS with 1% FCS at 4 °C in the dark until flow-cytometry analysis was started. All stained cells were analysed on a BD FACSCanto (BD Biosciences) flow cytometer. Cell death was always analysed in 10,000 EGFP-positive cells per sample using the FlowJo flow cytometry analysis software.

### Detection and identification of newly synthesized proteins

HeLa cells were grown at 37 °C and 5% CO_2_ in SILAC DMEM. SILAC media were essentially prepared as described previously^42^. Briefly, we used DMEM Glutamax lacking arginine and lysine (custom preparation from Gibco) supplemented with 10% dialyzed fetal bovine serum (dFBS, Gibco) for all experiments. To prepare ‘heavy' (H) or ‘medium' (M) SILAC media we added the following isotopically labelled amino acids: heavy, ^13^C_6_^15^N_4_ L-arginine and ^13^C_6_^15^N_2_ L-lysine; Medium, ^13^C_6_ L-arginine and ^2^H_4_ L-lysine. Labelled amino acids were purchased from Cambridge Isotope Laboratories. ‘Light' (L) SILAC medium was prepared by adding the corresponding non-labelled amino acids (Sigma).

Transient transfections of cells with plasmids were performed using linear polyethylenimine (Sigma). Twenty-four hours after transfection cells were washed thrice with pre-warmed PBS; then they were further cultivated for an hour in an appropriate cell culture medium lacking methionine. After 1 h cells were treated with 1 mM AHA (L-aziodohomoalanine, Anaspec) and grown for 4 h. The cells were trypsinized and collected for subsequent analysis.

Cells were lyzed according to the ‘Click-iT Enrichment Kit' protocol (Invitrogen) with minor modifications, as previously described^69^. In short, cells were incubated in Urea lysis buffer supplemented with complete protease inhibitors (Roche) and nuclease (Benzonase, Merck) on ice. Samples were spun down at 13,000*g* and supernatants were transferred to fresh Eppendorf tubes. Lysates were mixed 1:1:1 always with a EGFP control in either ‘light' or ‘heavy' present as a spike in. The spike was added so that all samples could be compared between the mass spec runs. Click reactions between newly synthesized azide-bearing proteins and alkyne-agarose beads were performed overnight following the kit instructions. Reduction of proteins by denaturation at 70 °C in the presence of 10 mM DTT was followed by alkylation of sulphydryl groups with 40 mM iodoacetamide were all executed according to the kit protocol. The alkyne beads were then stringently washed sequentially in SDS buffer, 8 M urea in 100 mM Tris (pH 8) and finally 80% acetonitrile by vortexing in buffer followed by centrifugation of beads and decanting of supernatant. AHA-containing proteins covalently linked to the alkyne beads were digested by LysC in 50 mM ammonium bicarbonate buffer with 5% acetonitrile for three hours and then by trypsin overnight. Resulting peptides were stored in StageTips^70^.

Peptides were eluted from StageTips by 80% acetonitrile and 0.1% formic acid and followed by evaporation of organic solvent. Peptides were resuspended in 5% acetonitrile and 3% trifluoroacetic acid and in this buffer loaded onto a 15-cm-long column with an inner diameter of 75 μm filled with ReproSil-Pur 120 C18-AQ 3 μm resin (Dr Maisch GmbH). Peptides were eluted from the column using a high-performance liquid chromatography system (ThermoScientific) by a four-hour gradient of increasing concentration of acetonitrile with a flow rate of 250 nl min^−1^. Eluted peptides were ionized by a heated electrospray ionization source (ThermoScientific) and analysed on a Q Exactive mass spectrometer (ThermoScientific). Ions in the MS-full scans were analysed in the orbitrap at a resolution of 70,000 after collecting 3,000,000 ions or after the maximum collection time of 20 ms. A data-dependent mode was used and the top 10 most intense ions in the full scan were selected for fragmentation in the higher-energy collision-induced dissociation cell. The resulting ion fragments were analysed in the orbitrap at a resolution of 17,500 after collecting 1,000,000 ions or a maximum collection time of 60 ms.

Raw files were processed using the MaxQuant software version 1.5.2.8 (ref. 71) with default settings except ‘match-between runs' which was activated. Arg10 and Lys8 were set as labels, carbamidomethyl of C-termini was set as fixed modification and N-terminal acetylation, deamidation of aspargine and glutamine and oxidation of methionine were set as variable modifications. The Andromeda search engine matched the acquired MS/MS spectra against an *in silico* trypsin/P digested human Uniprot data base (2014-01). False discovery rates were set to 1% both at peptide and protein levels and were assessed by in parallel searching against a reversed version of the database. Graphs and statistics were performed using R version 2.15.1 (R Foundation for Statistical Computing, Vienna, Austria).

### Protein transduction and confocal microscopy

Protein transduction was carried out as described previously with indicated modifications. Briefly, 4 × 10^5^ HeLa cells were plated onto glass-bottom dishes (Mattek) and on the next day cells were washed thrice with DMEM (Gibco). Flashed frozen recombinant proteins were thawed and briefly centrifuged at 20,000*g* on table top centrifuge at 4 °C. Cells were treated with recombinant proteins (25, 75 and 100 nM) in 800 μl of DMEM; after 4–5 h DMEM with 20% FCS containing penicillin (100 μg ml^−1^) and streptomycin (100 μg ml^−1^) was added to cells and they were incubated overnight. Next, live and dead cells were quantified in the EGFP-positive cell populations. Cellular uptake of recombinant proteins was monitored using confocal imaging microscopy. Cells were treated with 100 nM of recombinant proteins for 4 h, cells were washed with PBS, fixed with 4% formaldehyde. The cells were imaged using an Olympus Fluoview 1000 confocal laser scanning microscopy with a × 60/1.3 NA silicon oil lens. Images were acquired with a *z*-step size of 0.2 μm, a pinhole of 0.9 AU and line averaging of 48. The images were analysed using Fiji software.

### Static light scattering

A coupled RALS (Right-Angle Light Scattering)-refractive index detector (Malvern) was connected in-line to an analytical gel-filtration column Superdex S200 10/300 to determine absolute molecular masses of eluted proteins. Data were analysed with the provided software. The running buffer contained 20 mM HEPES (pH 7.5), 200 mM NaCl, 2 mM MgCl_2_ and 2 mM DTT. For each protein sample, 100 μl of a 3 mg ml^−1^ solution was applied.

### Isothermal titration calorimetry (ITC)

ITC experiments were performed using a VP-ITC titration microcalorimeter (GE Healthcare, Freiburg, Germany). All titrations were performed in a buffer containing 20 mM HEPES/NaOH pH 7.4, 200 mM NaCl, 5 mM MgCl_2_, 2 mM ADP at 25 °C. 80 μM of ASPL-C were titrated into 8 μM p97-ND1. Raw data in the form of incremental heat per mole of added ligand for the titration were fitted by nonlinear least squares with the ORIGIN7 software using a one-site binding model.

### Biolayer interferometry (BLI)

The binding kinetics of p97 with ASPL-His were measured by BLI using a single-channel BLItz instrument (Pall, fortéBio). 1 μM ASPL-His (20 mM HEPES/NaOH pH 7.4, 200 mM NaCl, 1 mg ml^−1^ BSA) was captured on HIS2 biosensors (Pall, fortéBio) for 400 s, and the biosensors were subsequently washed for 600 s in buffer (20 mM HEPES/NaOH pH 7.4, 200 mM NaCl). Subsequently, the kinetics were measured, beginning with a 60 s baseline, 200 s association phase with varying concentrations of p97 (20 mM HEPES/NaOH pH 7.4, 200 mM NaCl, 100 nM to 12.5 nM), and a 200 s dissociation phase in buffer. Negative control experiments with the HIS2 biosensor without ASPL-His against the maximally used concentration of p97 (100 nM) were carried out. Conversely, HIS2 biosensor loaded with ASPL-His against buffer control (20 mM HEPES/NaOH pH 7.4, 200 mM NaCl) were also carried out. All measurements were done at room temperature. The data were analysed using the programming language R using a 1:1 binding model.

### Data availability

The atomic coordinates of p97-ND1:ASPL-C and p97:ASPL-C_Δ_ have been deposited in the Protein Data Bank with accession codes 5IFS and 5IFW, respectively. All other data relevant to this work are available from the authors on reasonable request.

## Additional information

**How to cite this article:** Arumughan, A. *et al*. Quantitative interaction mapping reveals an extended UBX domain in ASPL that disrupts functional p97 hexamers. *Nat. Commun.* **7,** 13047 doi: 10.1038/ncomms13047 (2016).

## Supplementary Material

Supplementary Information Supplementary Figure 1 - 11 and Supplementary Table 1 - 2 and Supplementary References

Supplementary Movie 1 Different views of the p97-ND1:ASPL-C heterotetramer

Supplementary Movie 2 360° rotation of an isolated p97:ASPL-C? heterodimeric unit

Supplementary Movie 3 Different views of the p97:ASPL-C? heterotetramer

Supplementary Movie 4 Simulation of the re-orientation of the D2 domain in p97 protomers upon ASPL-C? binding
