# Supplementary material for: Quantitative interaction mapping reveals an extended UBX domain in ASPL that disrupts functional p97 hexamers
Source: Nat Commun. 2016 Oct 20;7:13047. doi: 10.1038/ncomms13047 (PMC5080433; doi:10.1038/ncomms13047)
Supplement: Supplementary Information — Supplementary Figure 1 - 11 and Supplementary Table 1 - 2 and Supplementary References [file ncomms13047-s1.pdf]

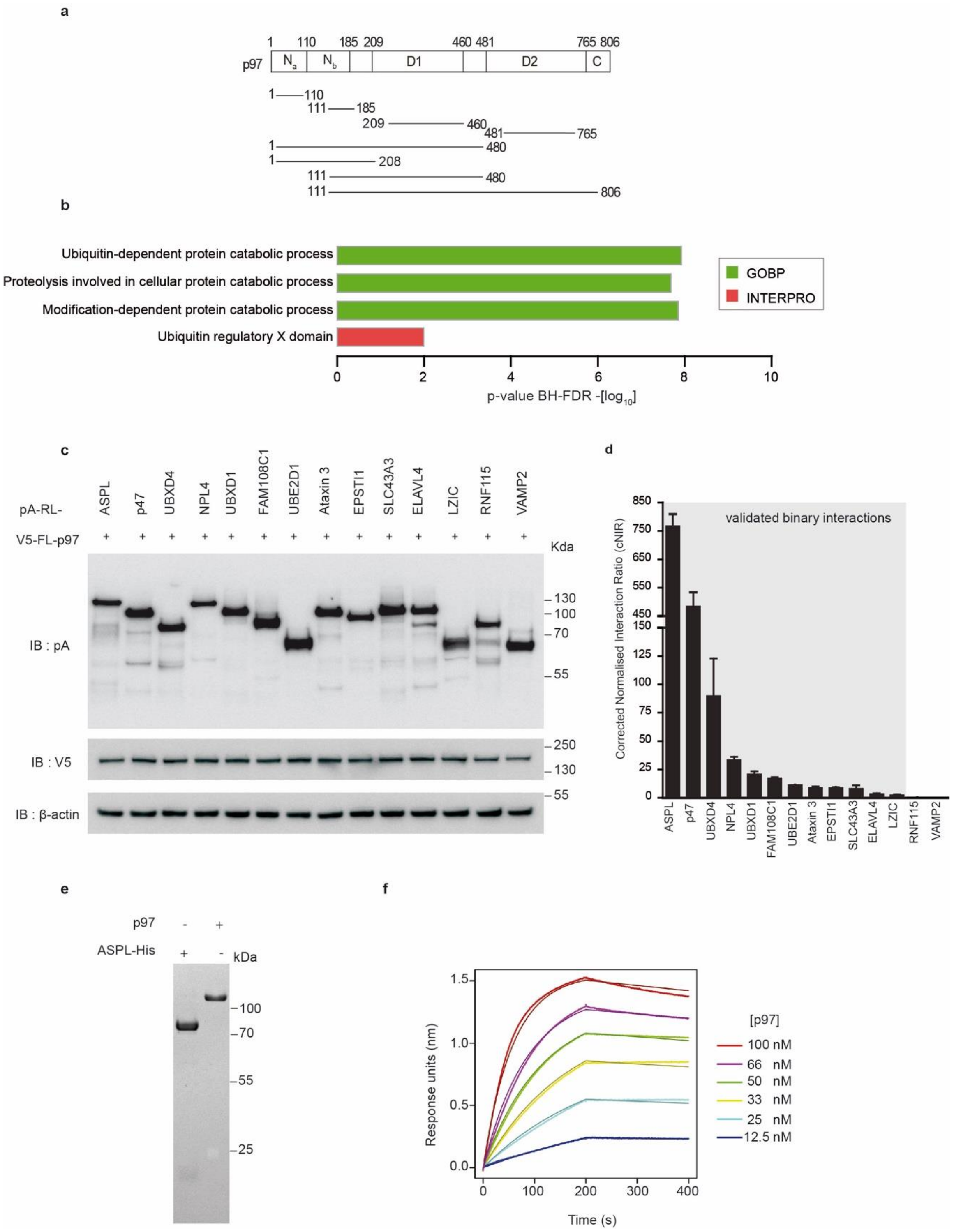

Supplementary Figure 1: Identification and functional analysis of p97 interacting proteins.

(**a**) A diagrammatic representation of the bait proteins used for automated yeast two-hybrid interaction screening**.** N_a and N_b are subdomains of the N domain in p97. D1 and D2 are conserved ATPase domains; C, C-terminal domain of p97.

(**b**) Biological processes enriched among the p97 binding partners were determined using the web-based functional annotation tool DAVID[1]. *P* values were calculated. Benjamini-Hochberg false discovery rate (BH-FDR); gene ontology biological processes (GOBP); Integrated resource of protein domains and functional sites (InterPro).

(**c**) Immunoblot showing the co-expression of fusion proteins in DULIP assays. Full-length p97-tagged with V5 Firefly luciferase (FL) was co-expressed with different p97 binding partners tagged with protein A-Renilla luciferase (pA-RL) in HEK293 cells. p97 was detected using an anti-V5 tag antibody, binding partners were identified using an anti-pA antibody.

(**d**) The p97:ASPL interaction showed the highest luminescence-based interaction ratio in DULIP assays.

(**e**) Coomassie-stained SDS-polyacrylamide gel showing the purified, recombinant full-length proteins p97 and ASPL-His applied for interaction studies.

(**f**) Biolayer interferometry sensogram showing the high-affinity interaction between full-length p97 and full-length ASPL-His. The recombinant C-terminally His-tagged ASPL protein immobilised to an anti-His antibody-coated biosensor shows binding to p97 in a concentration-dependent manner.

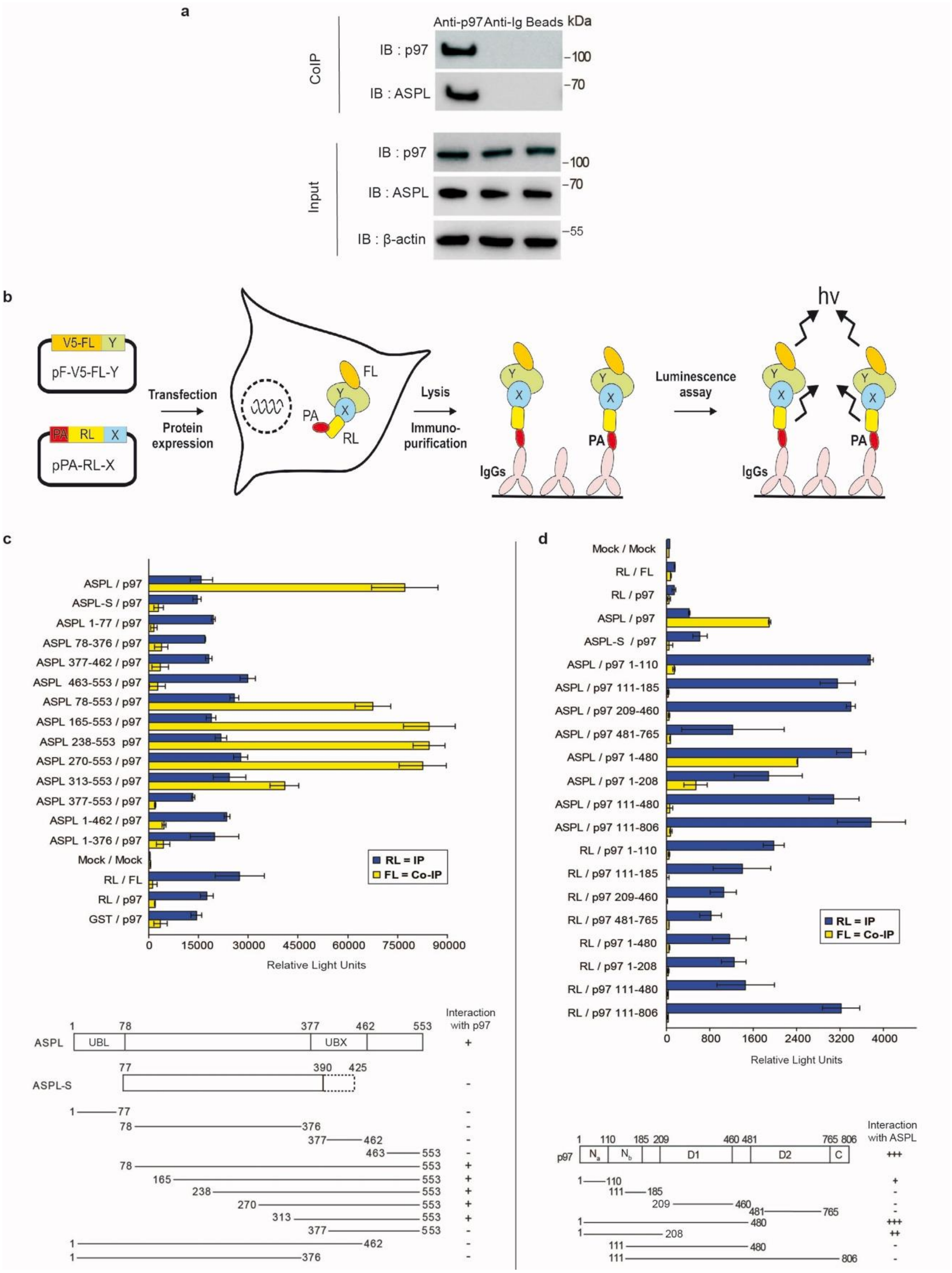

Supplementary Figure 2: Investigation of the interaction between ASPL and p97 using biochemical methods and DULIP assays.

(**a**) Co-immunoprecipitation of a p97:ASPL protein complex from human brain homogenate using an anti-p97 antibody. IB, immunoblotting; Anti-IgG antibody and beads alone were used as negative controls.

(**b**) Schematic representation of the dual luminescence-based co-immunoprecipitation (DULIP) experiments to detect protein-protein interactions in mammalian cells.

(**c**) Full-length ASPL (Uniprot ID Q9BZE9), the short isoform ASPL-S (Uniprot ID Q9BZE9-4) and various ASPL deletion fragments were co-expressed as protein A-tagged Renilla luciferase (RL) fusion proteins together with V5-tagged Firefly luciferase (FL) fused to p97 in HEK293 cells. Immunoprecipitation (blue bars) of ASPL fragments is shown. Co-immunoprecipitation of p97 was detected by quantification of FL activity (yellow bars). Data are expressed as mean ± SD of triplicates. Schematic summary of results from DULIP assays is shown below. Residues 390-425 represented as a dotted line indicate the amino acid sequence unique to ASPL-S.

(**d**) Various p97 fragments fused to V5-tagged Firefly luciferase (FL) were co-expressed with protein A- and Renilla luciferase (RL)-tagged ASPL in HEK293 cells. Immunoprecipitation (blue bars) of ASPL with IgG coated plates resulted in the co-precipitation of full-length p97 and various N-terminal fragments (yellow bars) in DULIP assays. Data are expressed as mean ± SD of triplicates. A schematic summary of the results is shown. ASPL most strongly interacts with residues 1-480 of p97 (p97-ND1). This fragment contains the N domain (residues

1-186), the D1 domain (residues 209-460) and the D1-D2 linker region (residues 460-480). The binding of ASPL to the N domain was weaker compared to the p97-ND1 fragment. ASPL interacted with the N_a subdomain (residues 1-110) but not with the N_b subdomain (residues 111-185) in DULIP assays.

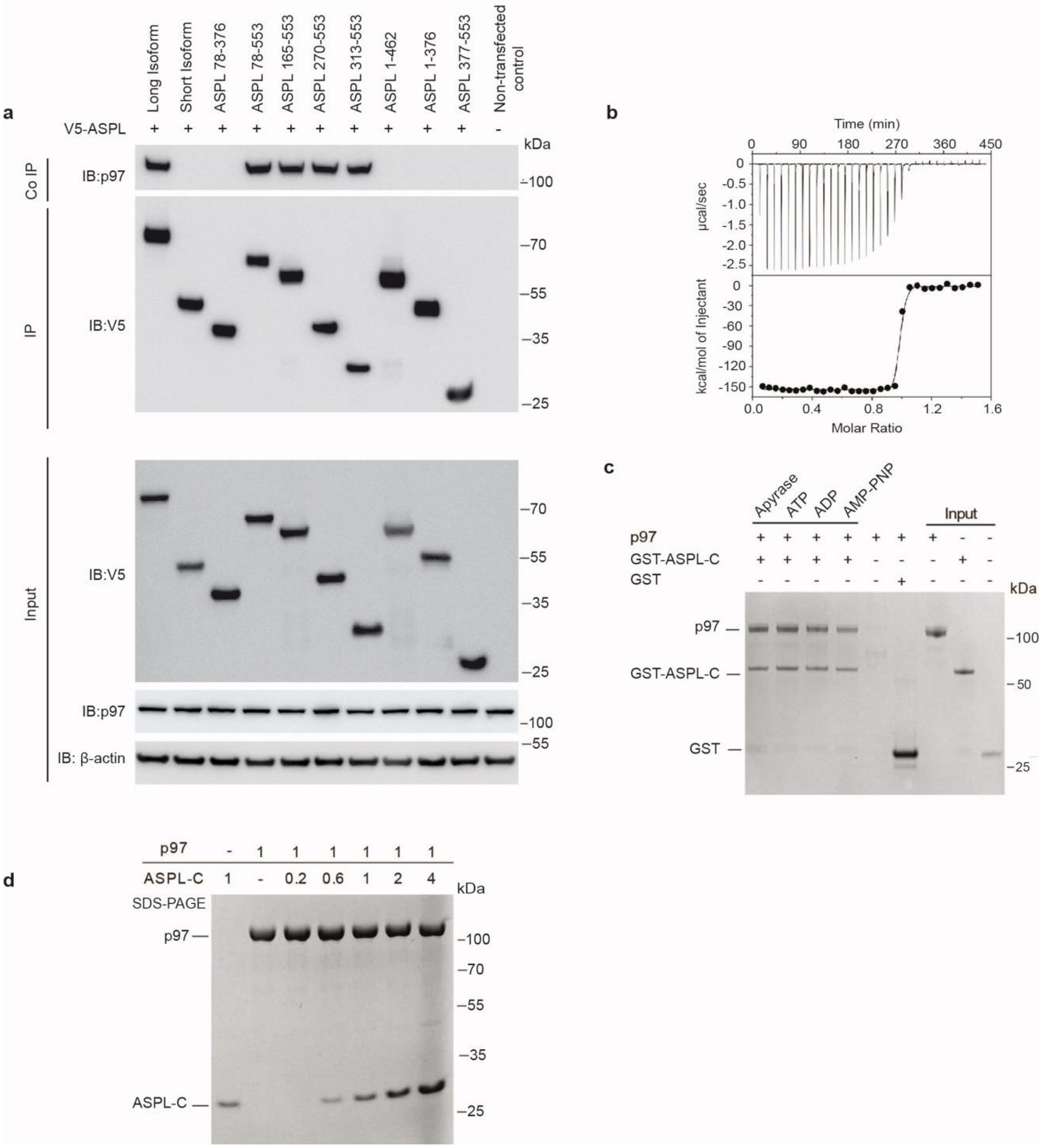

Supplementary Figure 3: Biochemical investigation of the interaction between p97 and ASPL

(**a**) Immunoblot showing the interaction of endogenous p97 with different ASPL fragments in co-immunprecipitation experiments. Full-length ASPL and truncated fragments thereof were overproduced in HEK293 cells as V5-tagged fusion

proteins. After 48h, ASPL containing protein complexes were immunoprecipitated using anti-V5 agarose beads. Co-precipitation of p97 was assessed using an anti-p97 antibody. Mock immunoprecipitation experiments with naïve cells were carried out as a negative control.

(**b**) Investigation of the interaction between ASPL-C and p97-ND1 using isothermal titration calorimetry (ITC). Purified recombinant proteins were used for this study. p97-ND1 pre-incubated with 2 mM ADP was titrated against ASPL-C containing 2 mM ADP. The data were fitted to a one-site binding model showing 1:1 stoichiometry between p97-ND1 and ASPL-C (n = 0.97, K_D = 0.2 nM, ΔH = -37 kcal/mol, ΔS = -79.5 cal/mol/deg).

(**c**) Coomassie-stained SDS-polyacrylamide gel showing the nucleotide-independent interaction of GST-ASPL-C with p97 examined by GST pull-down experiments.

(**d**) Coomassie-stained SDS-polyacrylamide gel showing recombinant full-length p97 and increasing amounts of ASPL-C.

a

p97:ASPL-C heterotetrameric complex

p97 (1-806 aa)

MASGADSKGDDLSTAILKQKNRPNRLIVDEAINEDNSVVSLSQPKMDELQLFRGDTVLLKGKKRREAVCIVLSDDTCSDEKIRMNRVVRNNLRV
RLGDVISIQPCPDVKYGKRIHVLPIDDTVEGITGNLFEVYLKPYFLEAYRPIRKGDIFLVRGGMRAVEFKVVETDPSPYCIVAPDTVIHCEGEP
IKREDEEESLNEVGYDDIGGCRKQLAQIKEMVELPLRHPALFKAIGVKPPRGILLYGPPGTGKTLIARAVANETGAFFFLINGPEIMSKLAGES
ESNLRKAFEEAEKNAPAIIFIDELDAIAPKREKTHGEVERRIVSQLLTLMDGLKQRAHVIVMAATNRPNSIDPALRRFGRFDREVDIGIPDATG
RLEILQIHTKNMKLADDVDLEQVANETHGHVGADLAALCSEAALQAIRKKMDLIDLEDETIDAEVMNSLAVTMDDFRWALSQSNPSALRETVVE
VPQVTWEDIGGLEDVKRELQELVQYPVEHPDKFLKFGMTPSKGVLFYGPPGCGKTLLAKAIANECQANFISIKGPELLTMWFGESEANVREIFD
KARQAAPCVLFFDELDSIAKARGGNIGDGGGAADRVINQILTEMDGMSTKKNVFIIGATNRPDIIDPAILRPGRLDQLIYIPLPDEKSRVAILK
ANLRKSPVAKDVDLEFLAKMTNGFSGADLTEICQRACKLAIRESIESEIRRERERQTNPSAMEVEEDDPVPEIRRDHFEEAMRFARRSVSDNDI
RKYEMFAQTLQQSRGFGSFRFPSGNQGGAGPSQGSGGGTGGSVYTEDNDDDLYG

ASPL-C (313-553 aa)

VDREPVDREPVVCHPDLEERLQAWPAELPDEFFELTVDDVRRRLAQLKSERKRLEEAPLVTKAFREAQIKEKLERYPKVALRVLFPDRYVLQGF
FRPSETVGDLRDFVRSHLGNPELSFYLFITPPKTVLDDHTQTLFQANLFPAALVHLGAEEPAGVYLEPGLLEHAISPSAADVLVARYMSRAAGS
PSPLPAPDPAPKSEPAAEEGALVPPEPIPGTAQPVKRSLGKVPKWLKLPASKR

b

p97:ASPL-C heterooctomeric complex

p97 (1-806 aa)

MASGADSKGDDLSTAILKQKNRPNRLIVDEAINEDNSVVSLSQPKMDELQLFRGDTVLLKGKKRREAVCIVLSDDTCSDEKIRMNRVVRNNLRV
RLGDVISIQPCPDVKYGKRIHVLPIDDTVEGITGNLFEVYLKPYFLEAYRPIRKGDIFLVRGGMRAVEFKVVETDPSPYCIVAPDTVIHCEGEP
IKREDEEESLNEVGYDDIGGCRKQLAQIKEMVELPLRHPALFKAIGVKPPRGILLYGPPGTGKTLIARAVANETGAFFFLINGPEIMSKLAGES
ESNLRKAFEEAEKNAPAIIFIDELDAIAPKREKTHGEVERRIVSQLLTLMDGLKQRAHVIVMAATNRPNSIDPALRRFGRFDREVDIGIPDATG
RLEILQIHTKNMKLADDVDLEQVANETHGHVGADLAALCSEAALQAIRKKMDLIDLEDETIDAEVMNSLAVTMDDFRWALSQSNPSALRETVVE
VPQVTWEDIGGLEDVKRELQELVQYPVEHPDKFLKFGMTPSKGVLFYGPPGCGKTLLAKAIANECQANFISIKGPELLTMWFGESEANVREIFD
KARQAAPCVLFFDELDSIAKARGGNIGDGGGAADRVINQILTEMDGMSTKKNVFIIGATNRPDIIDPAILRPGRLDQLIYIPLPDEKSRVAILK
ANLRKSPVAKDVDLEFLAKMTNGFSGADLTEICQRACKLAIRESIESEIRRERERQTNPSAMEVEEDDPVPEIRRDHFEEAMRFARRSVSDNDI
RKYEMFAQTLQQSRGFGSFRFPSGNQGGAGPSQGSGGGTGGSVYTEDNDDDLYG

ASPL-C(313-553 aa)

VDREPVDREPVVCHPDLEERLQAWPAELPDEFFELTVDDVRRRLAQLKSERKRLEEAPLVTKAFREAQIKEKLERYPKVALRVLFPDRYVLQGF
FRPSETVGDLRDFVRSHLGNPELSFYLFITPPKTVLDDHTQTLFQANLFPAALVHLGAEEPAGVYLEPGLLEHAISPSAADVLVARYMSRAAGS
PSPLPAPDPAPKSEPAAEEGALVPPEPIPGTAQPVKRSLGKVPKWLKLPASKR

c

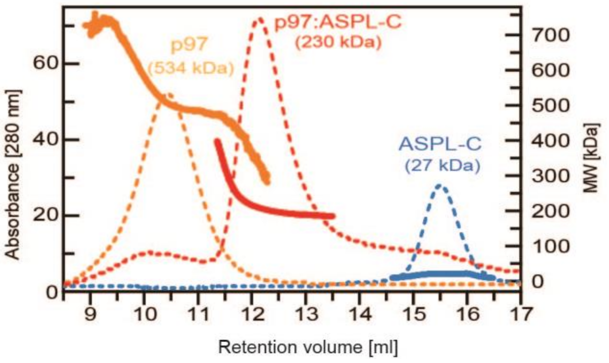

Supplementary Figure 4: ASPL-C converts p97 hexamers into stable p97:ASPL-C heterotetramers.

(**a-b**) Analysis of p97:ASPL-C protein bands prepared from BN gels by mass spectrometry**.** p97:ASPL-C complexes migrating at ~230 or ~480 kDa were excised from Coomassie-stained BN gels and subjected to in-gel tryptic digestion. The composition of the peptides was analyzed by liquid chromatography-mass spectrometry (LC-MS). The peptides identified from the protein bands are highlighted in red. The identification of peptides of ASPL and p97 in both protein bands (~230 and ~480 kDa) suggests the formation of stable protein complexes.

(**c**) p97 (orange), ASPL-C (blue) and a 1:1 mixture of both proteins (red) were resolved by size-exclusion chromatography (SEC) using a Superdex 200 10/300 column. Static light scattering in line with the SEC column showed that p97 (534 kDa) is a hexamer, ASPL-C (27 kDa) is a monomer and p97:ASPL-C is a heterotetrameric protein complex (230 kDa). The molecular masses determined by static light scattering (indicated above the respective peaks in the chromatogram) were in agreement with the theoretically calculated molecular masses of the respective proteins or complexes indicated.

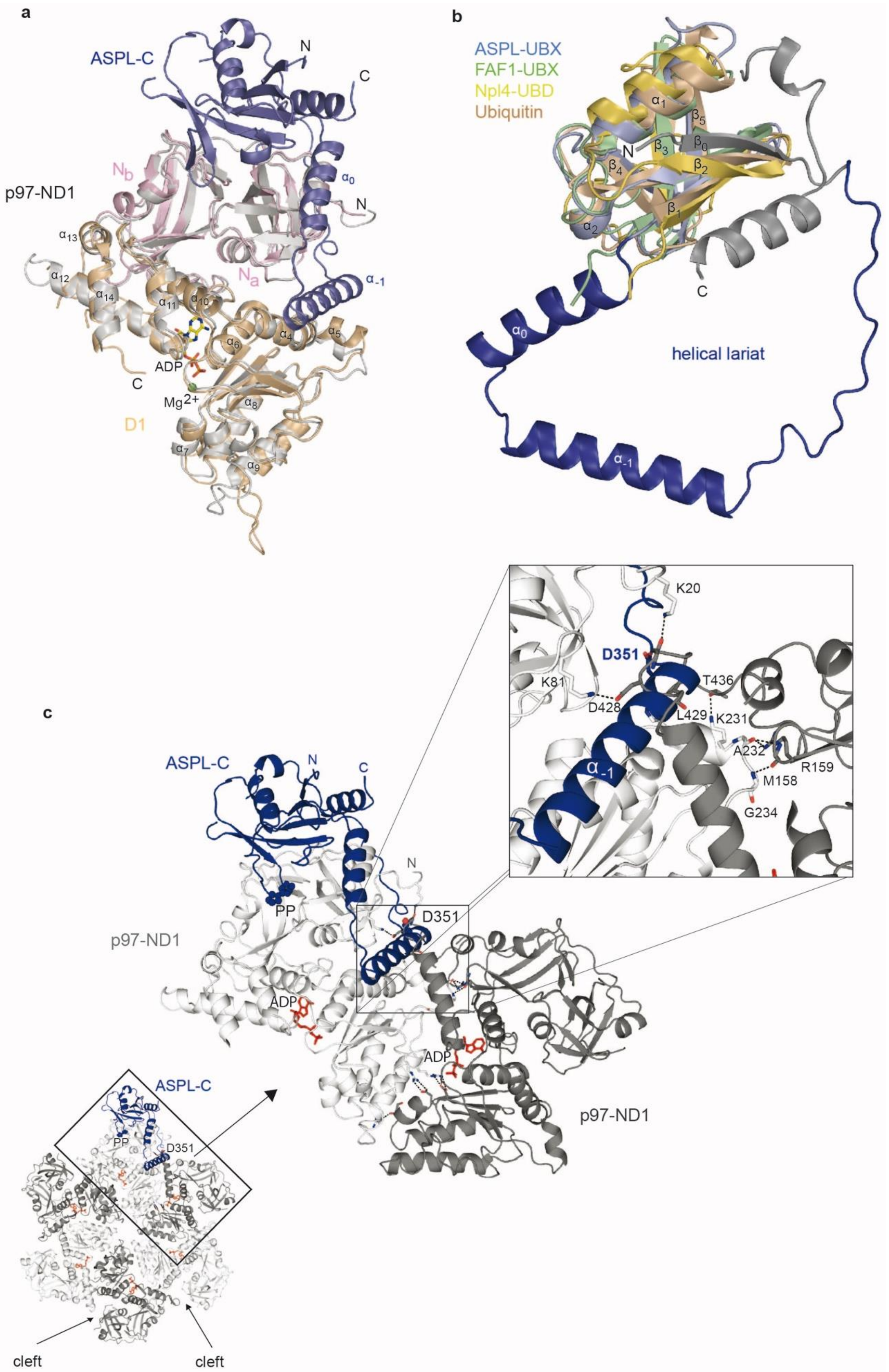
a
ASPL-C
N
C
p97-ND1
N_b
α_0
N
α_{13}
α_{12}
α_{14}
α_{11}
α_{10}
N_a
α_{-1}
α_4
α_5
α_6
ADP
C
Mg^{2+}
α_8
D1
α_7
α_9
b
ASPL-UBX
FAF1-UBX
Npl4-UBD
Ubiquitin
α_1
β_5
β_3
β_0
N
β_4
β_2
β_1
α_2
C
α_0
helical lariat
α_{-1}
c
K20
D351
K81
T436
D428
L429
K231
A232
R159
α_{-1}
M158
G234
ASPL-C
N
C
N
PP
D351
p97-ND1
ADP
ADP
ASPL-C
p97-ND1
PP
D351
cleft
cleft

Supplementary Figure 5: The α_{-1} helix in the ASPL-C lariat structure targets the hexameric assembly interface of p97.

(**a**) Superposition of the structures of p97-ND1:p47 (PDB 1S3S colored in grey; for clarity the p47 molecule was removed) and of p97-ND1:ASPL-C (p97-N domain in salmon color, D1 domain in wheat color, ASPL-C in blue). The bound ADP molecule is depicted with atom-colored sticks and the Mg^{2+} ion as a green sphere.

(**b**) Superposition of the eUBX domain of ASPL (light blue) with the UBX domains of FAF1 (PDB 3QQ8, green; rmsd = 1.14 Å), Npl4 (PDB 2PJH, yellow; rmsd = 2.22 Å) and ubiquitin (PDB 1UBI, wheat; rmsd = 1.19 Å), all shown in ribbon representations. The helical lariat of ASPL-C is shown in dark blue, and the UBX extensions are highlighted in gray.

(**c**) p97-ND1 hexamer (PDB: 1S3S) with adjacent protomers represented in two shades of grey. ASPL-C (blue) as observed in the p97-ND1:ASPL-C structure was superimposed onto one protomer of the p97-ND1 hexamer. Arrows indicate the preformed clefts between the protomers in the p97 hexamer, which are targeted by the helical lariat structure of ASPL. The magnified view shows a steric clash of the α_{-1} helix of ASPL with the interprotomeric interface of the p97 hexamer.

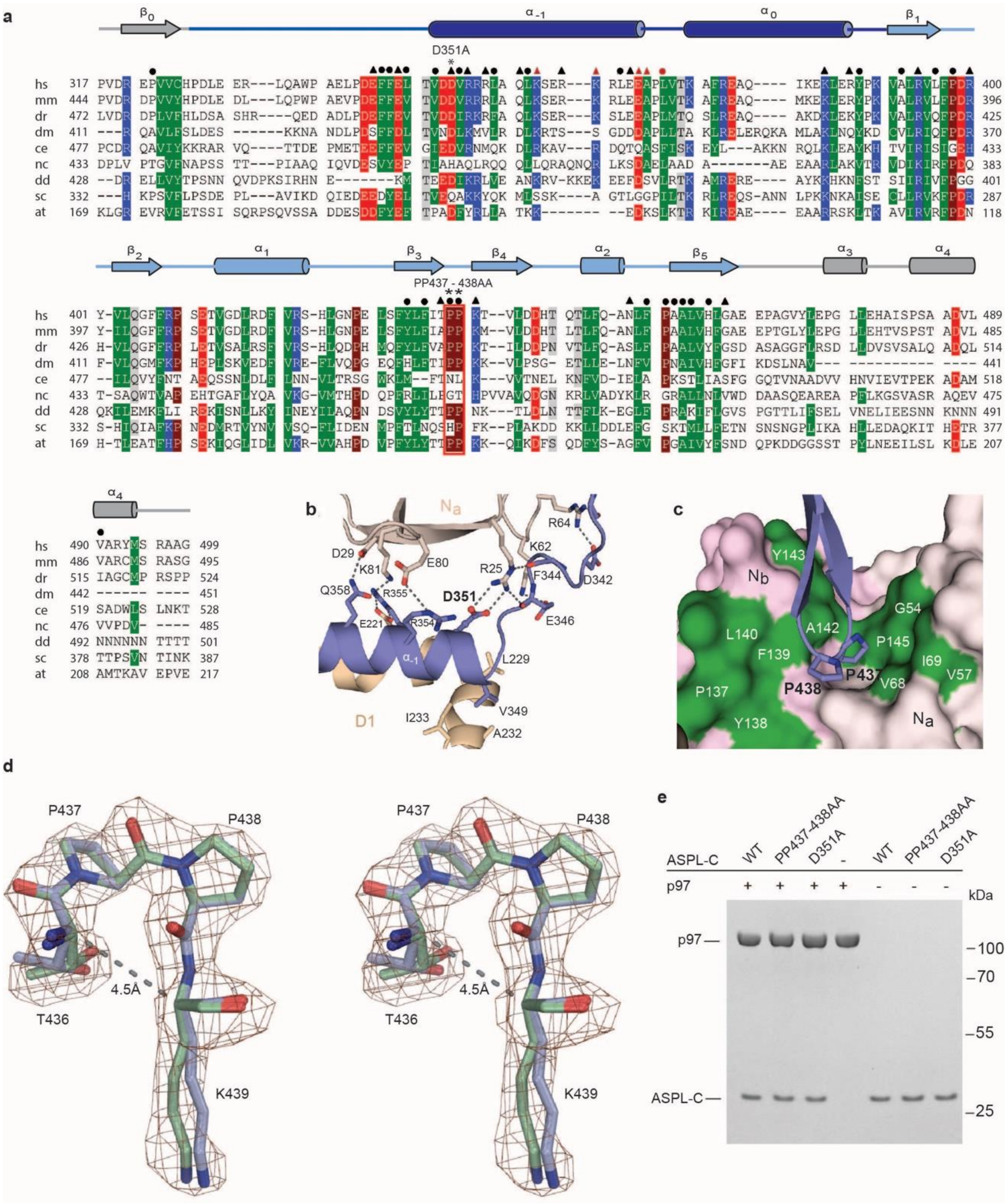
a
β0
α-1
α0
β1
D351A
hs 317 PVDR EPVVCHPDLE ER---LQAWP AELPDEFFEL TVDDVRRRLA QLKSER---K RLEEAPLVTK AFREAQ---- IKEKLERYPK VALRVLFPDR 400
mm 444 PVDR DPVVYHPDLE DL---LQPWP AEVPDEFFEV TVDDVRRRLA QLKSER---K RLEEAPLVTK AFREAQ---- MKEKLERYPK VALRVLFPDR 396
dr 472 LVDR DPLVFHLDSA SHR----QED ADLPDEFFEV TVDDIRKRFA QLKSER---K SLEEAPLMTQ SLREAQ---- AKDKLEKYPK VALRVQFPDR 425
dm 411 ---R QAVLFSLDES -------KKNA NDLPDSFFDL TVNDLKMVLR DLRRTS---S GDDDAPLLTA KLRELERQKA MLAKLNQYKD CVLRIQFPDR 370
ce 477 PCDR QAVIYKKRAR VQ----TTDE PMETEEFFEV GIEDVRNMQK DLRKAV---R DQTQASFISK EYL---AKKN RQLKLEAYKH TVIRISIGEH 433
nc 433 DPLV PTGVFNAPSS TT---PIAAQ IQVDESVYEP TIAHAQLRQQ QLLQRAQNQR LKSDAELAAD A-------AE EAARLAKVTR VDIKIRFPDQ 383
dd 428 --DR ELLVYTPSNN QVDPKSIRHN E-------KM TEEDIKRLVE ANKRV-KKEK EEFDSVLRTK AMRERE---- AYKKHKNFST SIIRIVFPGG 401
sc 332 ---H KPSVFLPSDE PL---AVIKD QIEDEEDYEL TVEQAKKYQK MLSSK----A GTLGGPILTK RLREQS-ANN LPKKNKAISE CLLRVKFPDR 287
at 169 KLGR EVRVFETSSI SQRPSQVSSA DDESDDFYEF TPADFYRLIA TKT------- --EDKSLKTR KIREAE---- EAARRSKLTK AVIRVRFPDN 118
β2
α1
β3
β4
α2
β5
α3
α4
PP437 - 438AA
hs 401 Y-VLQGFFRP SETVGDLRDF VRS-HLGNEE LSFYLFITPP KT--VLDDHT QTLFQ-ANLF PAALVHLGAE EPAGVYLEPG LLEHAISPSA ADVL 489
mm 397 Y-ILQGFFRP SETVGDLRDF VRS-HLGNEE LSFYLFIAPP KM--VLDDHT LTLEQ-ANLF PAALVHFGAE EPTGLYLEPG LLEHTVSPST ADVL 485
dr 426 H-VLQGFFRP LETVSALRSF VRS-HLQDEH MQFYLFVAPP KT--ILDDPN VTLEQ-ASLF PAALVYFGSD ASAGGFLRSD LLDVSVSALQ ADQL 514
dm 411 F-VLQGMFKP HEPLSKVEDF VRE-FLVQEG EQFHLFTIPP KK--VLPSG- ETLLE-LNFV PNAIVHFGFI KDSLNAV--- ---------- ---- 441
ce 477 --ILQVYFNT AEQSSNLDLF INN-VLTRSG WKLM--FTNL K---VVTNEL KNFVD-IELA PKSTLIASFG GQTVNAADVV HNVIEVTPEK ADAM 518
nc 433 T-SAQWTVAP EHTGAFLYQA VKG-VMTHED QPFRLILEGT HPVVAVQDGN KRLVADYKLR GRALINLVWD DAASQEAREA PFLKGSVASR AQEV 475
dd 428 QKILEMKFLI RDKISNLLKY INEYILAQEN DSVYLYTTPP NK---TLDLN TTFLK-EGLF FRAKIFLGVS PGTTLIFSEL VNELIEESNN KNNN 491
sc 332 S-HIQIAFKP NEDVRTVYNV VSQ-FLIDEN MPFTLNQSHP FK--PLAKDD KKLIDDLEFG SKTMLLFETN SNSNGPLIKA HLLEDAQKIT HETR 377
at 169 H-TLEATFHP SPKIQGLIDL VKR-VVAHED VPFYLYTTPP KK--QIKDFS QDFYS-AGFV PGAIVYFSND QPKDDGGSST PYLNEEILSL KDLE 207
α4
hs 490 VARYMS RAAG 499
mm 486 VARCMS RASG 495
dr 515 IAGCMP RSPP 524
dm 442 ------ ---- 451
ce 519 SADWLS LNKT 528
nc 476 VVPDV- ---- 485
dd 492 NNNNNN TTTT 501
sc 378 TTPSVN TINK 387
at 208 AMTKAV EPVE 217
b
Na
R64
K62
D29
E80
R25
K81
F344
D342
Q358
R355
D351
R354
E221
E346
α-1
L229
V349
I233
D1
A232
c
Y143
Nb
G54
L140
A142
P145
F139
I69
V57
P437
V68
P137
P438
Na
Y138
d
P437
P438
4.5Å
T436
K439
e
ASPL-C
WT
PP437-438AA
D351A
-
p97
+
kDa
p97
100
70
55
ASPL-C
25

Supplementary Figure 6: The eUBX domain of ASPL is evolutionary conserved.

(**a**) Structure-based sequence alignment of ASPL orthologues. Amino acid sequences of ASPL from *Homo sapiens* (hs; Uniprot ID Q9BZE9), *Mus musculus* (mm; Uniprot ID Q8VBT9), *Danio rerio* (dr; Uniprot ID Q568S9), *Drosophila melanogaster* (dm; Uniprot ID Q6NL44), *Caenorhabditis elegans* (ce; Uniprot ID Q17425), *Neurospora crassa* (nc; Uniprot ID Q7SEP0), *Dictyostelium discoideum* (dd; Uniprot ID Q54X06), *Saccharomyces cerevisiae* (sc; Uniprot ID P54730) and *Arabidopsis thaliana* (at; Uniprot ID Q9LK22) were aligned by Clustal Omega and manually adjusted. Residues with a conservation of greater than 60% are color-coded (D, E in red; R, K, H in blue; N, Q, S, T in gray; L, I, V, F, Y, W, M, C in green; P, G in brown). α-Helices are shown as cylinders and β-strands as arrows. Residues contributing to p97 binding (heterodimer) are indicated by (▲) for charged and by (●) for van der Waals interactions. Mutations are shown by (*) on top of the alignment. ASPL residues involved in heterotetramer assembly interface formation are marked by (▲) for charged and (●) for van der Waals interactions.

(**b**) Close-up view of the N-terminus of the α_{-1} helix of ASPL-C (blue) located between the N_a and D1 domains of p97-ND1, highlighting residue Asp351.

(**c**) Surface representation of the binding pocket on the p97 N_a (salmon color) and N_b (pink) subdomains. The eUBX domain of ASPL-C with the Pro437/Pro438 loop is shown in blue, and hydrophobic residues of p97 are colored in green.

(**d**) Stereo image of the *cis*-Pro touch-turn motif. Superposition of the *cis*-Pro touch-turn motif in the β_3/β_4 loop of ASPL-C. Residues belonging to the two ASPL-C molecules in the heterotetrameric p97-ND1:ASPL-C complex are shown in stick representation with green and light blue carbon atoms, respectively. The electron density ($2F_o$-F_c map) is contoured at 1.0σ, and the distance between $C\alpha^i$ and $C\alpha^{i+3}$ is indicated with a dashed line.

(**e**) Coomassie-stained SDS-polyacrylamide gel showing recombinant full-length p97 and indicated variants of ASPL-C.

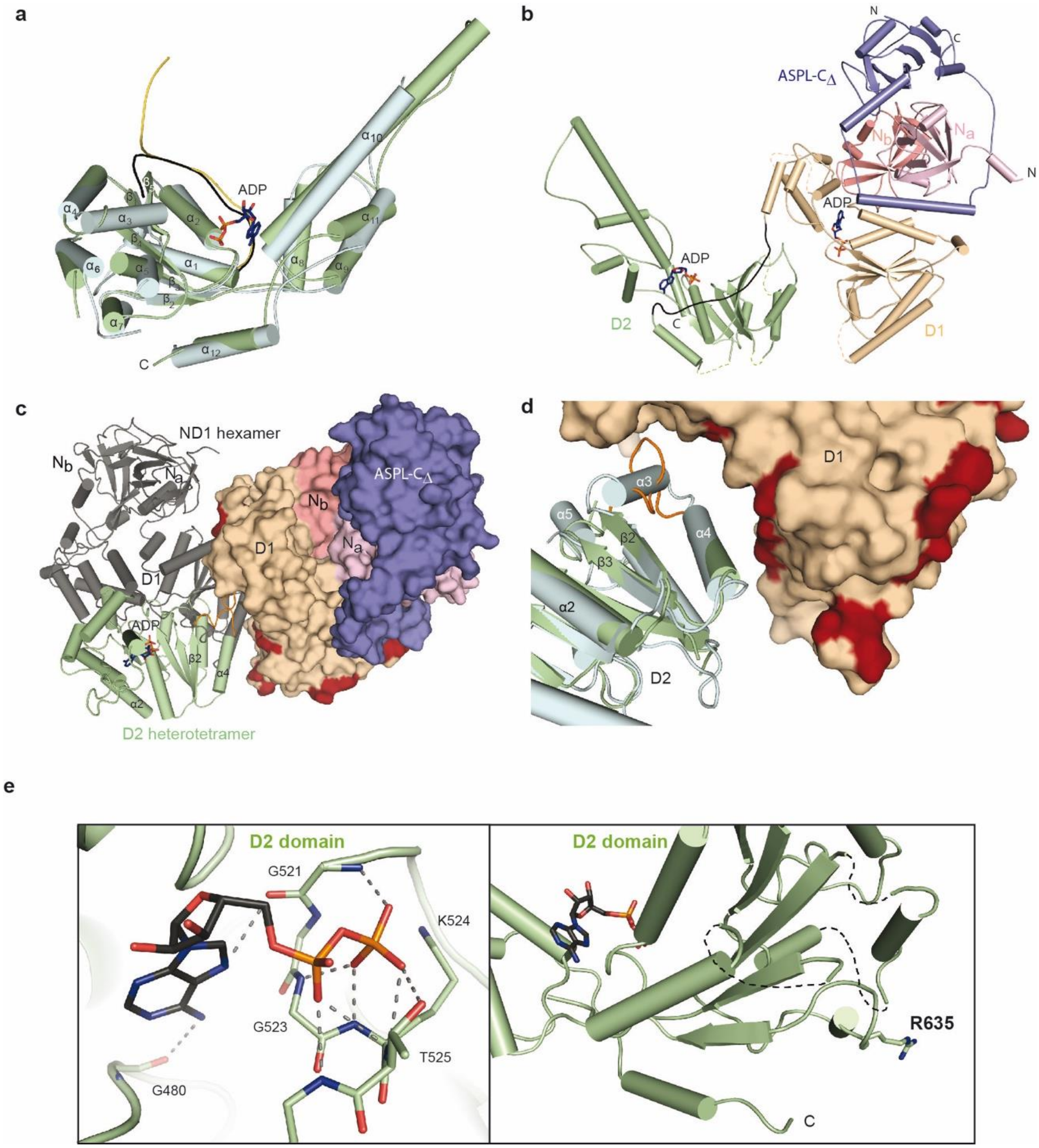

Supplementary Figure 7: Formation of the p97:ASPL-C_Δ heterotetramer is accompanied by a delocalization of the D2 ATPase domain in p97.

(**a**) Superposition of the p97 D2 domain as present in p97 hexamers (PDB: 3CF1, light blue) or in complex with ASPL-C_Δ (green) shows only slight changes

(rmsd = 1.83 Å) in its secondary or tertiary structure. The significant differences are in the D1-D2 linker region, which is shown in yellow for the hexameric p97 D2 domain and in black for the D2 domain present in the complex with ASPL-C_{Δ}.

(**b**) An isolated p97:ASPL-C_{Δ} heterodimeric unit with the p97 N domain (pink), the D1 domain (wheat color), the D2 domain (green) and ASPL-C_{Δ} in blue is shown as a cartoon representation.

(**c**) A heterodimeric unit of the p97:ASPL-C_{Δ} heterotetramer is shown as molecular surface using colors as in (d). The red surface depicts residues that are involved in interprotomer interface formation within p97 hexamers (PDB: 3CF1). Bound ADP is shown as a stick model. One adjacent p97-ND1 protomer of the hexamer is presented in gray, and the novel position of the D2 domain in the p97:ASPL-C_{Δ} heterodimer unit as green cartoon. Overall, the re-oriented D2 domain in the heterotetrameric p97:ASPL-C_{Δ} complex partially overlaps the space occupied by the adjacent D1 domain in the p97 hexamer.

(**d**) Magnified view at the p97-D2 domain orientation (green) with respect to the D1 domain in the p97:ASPL-C_{Δ} complex, similar as in (c). A p97-D2 domain found in the hexamer (light blue) is superimposed as in (a). In the p97:ASPL-C_{Δ} complex, helix 3 of the p97-D2 domain is transformed into a loop conformation (orange) and fills, together with helix 4, the vacant space of the hexamer D1 interface by partially shielding the exposed interprotomer interfacing residues.

(**e**) Details of the nucleotide binding pocket of the p97:ASPL-C_{Δ} D2 domain. The ADP molecule and all residues involved in nucleotide binding are shown in a

stick representation. The left panel represent detailed views of the nucleotide binding sites, and the right panel show the new spatial location of arginine finger relative to the nucleotide binding sites in ASPL-C_{Δ} bound p97 structure. D2 (green) domain with bound ADP molecule in the p97:ASPL-C_{Δ} complex. Inspection of the nucleotide-binding pocket revealed that ADP binding to the p97 D2 domains is unchanged after disassembly by ASPL. In heterotetramers the same p97 residues are involved in ADP binding as in the published hexameric p97 structure[2], except for the arginine finger (Arg 635), which no longer contributes to ADP binding due to its different spatial position in the heterotetramer. Arg 635 in the D2 domain, which is critical for the ATPase activity in hexamers[3], is solvent-exposed after heterotetramerization.

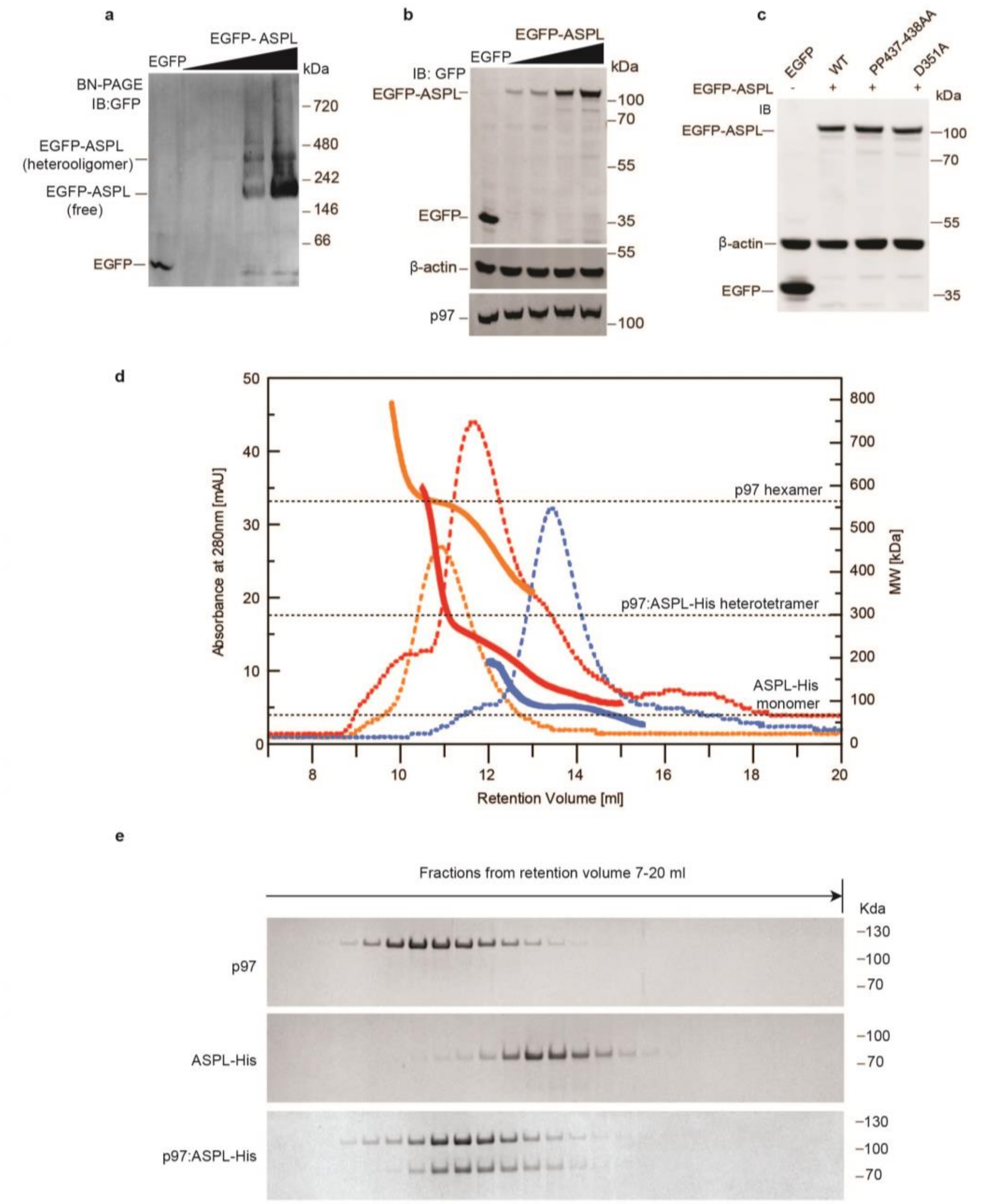

Supplementary Figure 8: Full-length ASPL remodels the architecture of p97 hexamers.

(**a**) Immunoblot showing the analysis of protein extracts prepared from HEK293 cells by BN-PAGE. The formation of EGFP-ASPL:p97 heterooligomers was monitored using an anti-GFP antibody.

(**b**) SDS-PAGE and immunoblotting experiments show the overproduction of EGFP-ASPL in HEK293 cells in a concentration-dependent manner. EGFP-ASPL was detected using an anti-GFP antibody. β-Actin expression was used as a loading control. The endogenous p97 protein was detected using an anti-p97 antibody.

(**c**) Immunoblot showing the expression of EGFP-ASPL and its mutant variants in HEK293 cells. The expression of recombinant proteins was detected using an anti-GFP antibody. Expression of β-actin was used as a loading control.

(**d-e**) p97, ASPL-His and a 1:1 mixture of both proteins were resolved by size-exclusion chromatography (SEC, dotted line) using a Superdex 200 10/300 column. Static light scattering in line with the SEC column (continuous line) showed that p97 (orange ~530 kDa) is a hexamer, ASPL-His (blue ~75 kDa) is a monomer and p97:ASPL-His is a heterotetrameric protein complex (red ~300 kDa). Expected molecular weights of proteins are represented using dotted black lines

(**e**) Coomassie-stained SDS-polyacrylamide gel showing elution profiles of recombinant proteins.

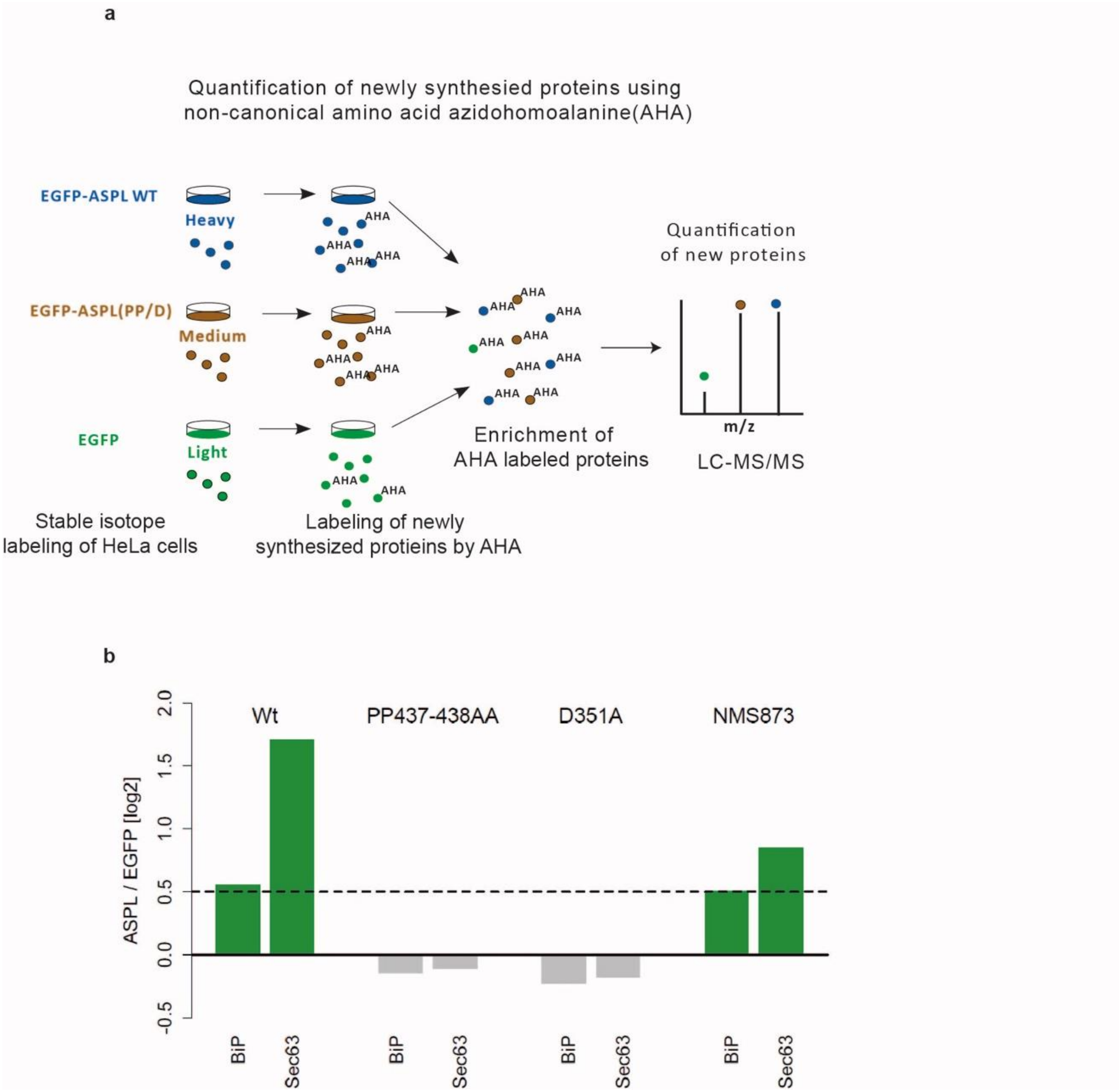

Supplementary Figure 9: Overproduction of ASPL in HeLa cells alters the abundance of proteins that play a role the ERAD pathway.

(**a**) Schematic representation of the quantitative analysis of newly synthesized proteins that were metabolically labeled with the non-canonical amino acid azidohomoalanine (AHA).

(**a**-**b**) EGFP-tagged ASPL WT and indicated variants were overproduced in stable isotope-labeled HeLa cells for 24h. Then, the cells were treated with AHA for 4h for metabolically labelling of the newly synthesized proteins. The AHA labeled proteins were captured by click chemistry-based pull down experiments and identified by quantitative SILAC-based mass spectrometry (LC-MS/MS). Fold changes in the protein amounts of ASPL WT and the indicated variants PP (PP437-438AA) and D (D351A) are shown.

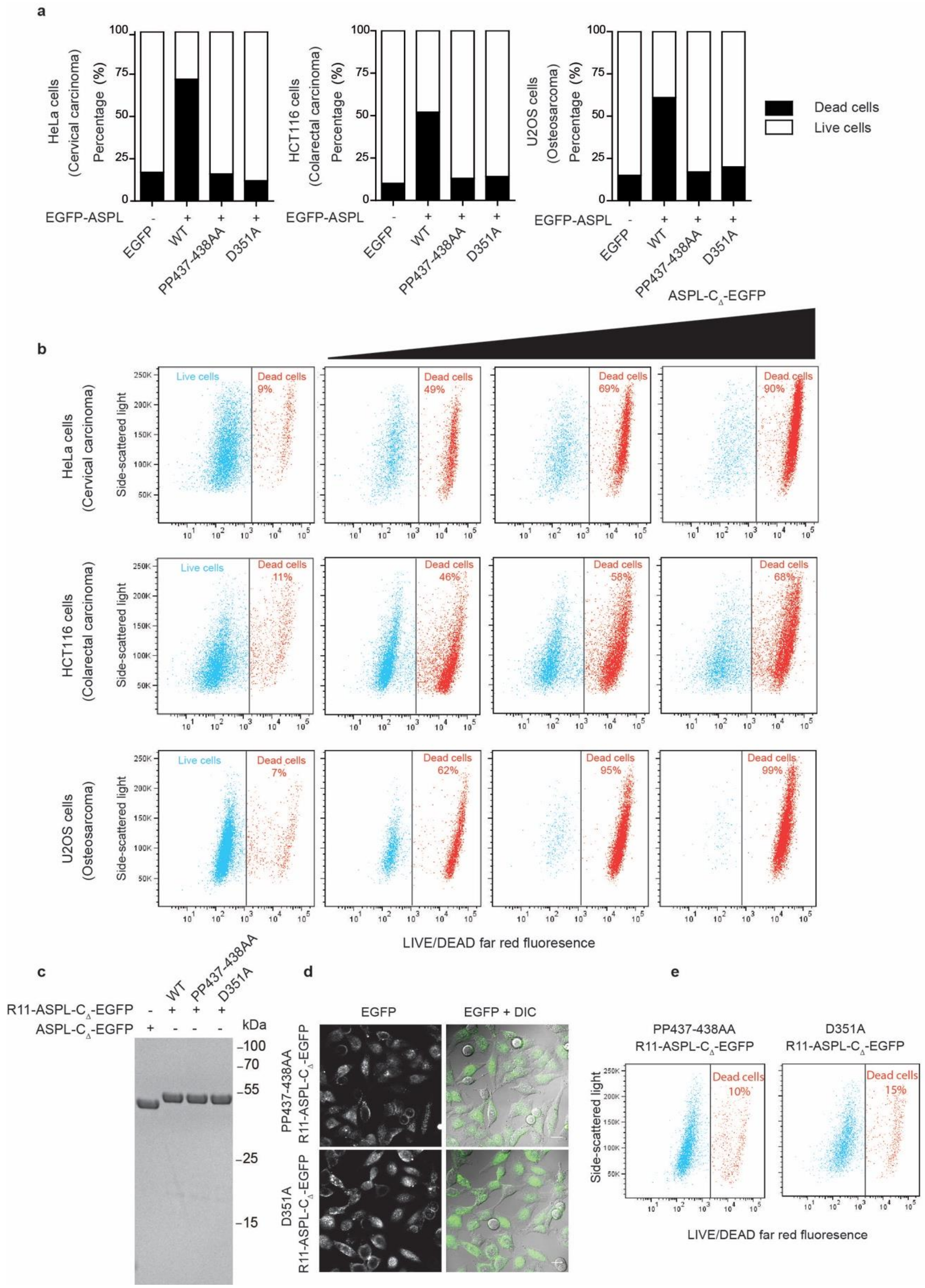
a
HeLa cells (Cervical carcinoma) Percentage (%)
HCT116 cells (Colarectal carcinoma) Percentage (%)
U2OS cells (Osteosarcoma) Percentage (%)
Dead cells
Live cells
EGFP-ASPL
EGFP
WT
PP437-438AA
D351A
ASPL-C$_\Delta$-EGFP
b
Side-scattered light
Live cells
Dead cells
9%
49%
69%
90%
11%
46%
58%
68%
7%
62%
95%
99%
LIVE/DEAD far red fluoresence
c
R11-ASPL-C$_\Delta$-EGFP
ASPL-C$_\Delta$-EGFP
kDa
100
70
55
25
15
d
EGFP
EGFP + DIC
PP437-438AA R11-ASPL-C$_\Delta$-EGFP
D351A R11-ASPL-C$_\Delta$-EGFP
e
PP437-438AA R11-ASPL-C$_\Delta$-EGFP
D351A R11-ASPL-C$_\Delta$-EGFP
10%
15%

Supplementary Figure 10: Overproduction of an ASPL fragment harboring the eUBX domain induces cell death in various cancer cell lines.

(a) Overproduction of EGFP-tagged WT ASPL but not its mutant variants potently induce mammalian cell death. Cells were stained with a LIVE/DEAD fluorescent dye. Then, live and dead cell populations were quantified using flow cytometry.

(**b**) EGFP-ASPL-C_{Δ} was overproduced in a concentration dependent manner in the indicated cancer cell lines. After 72 h, cells were stained with a LIVE/DEAD fluorescent dye and finally live and dead cell populations were quantified using flow cytometry.

(**c**) Coomassie-stained SDS-polyacrylamide gel showing the indicated ASPL-C_{Δ} recombinant proteins that were applied for the treatment of mammalian cells.

(**d**) Confocal imaging micrographs of fixed HeLa cells showing the uptake of the indicated recombinant R11-ASPL-C_{Δ}-EGFP fusion proteins into cells. Scale bar 10 µm.

(**e**) HeLa cells were treated for 24 h with the indicated, mutated recombinant proteins and stained with a LIVE/DEAD fluorescent dye. Finally, live and dead cell populations were quantified using flow cytometry.

Fig. 2c

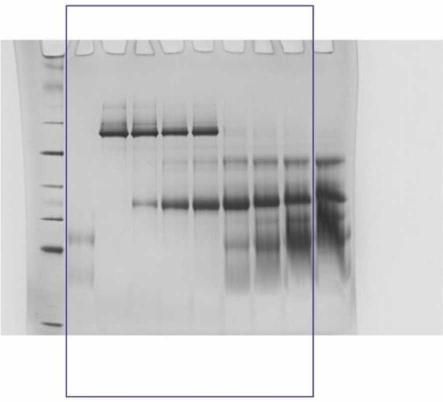

Fig. 3e

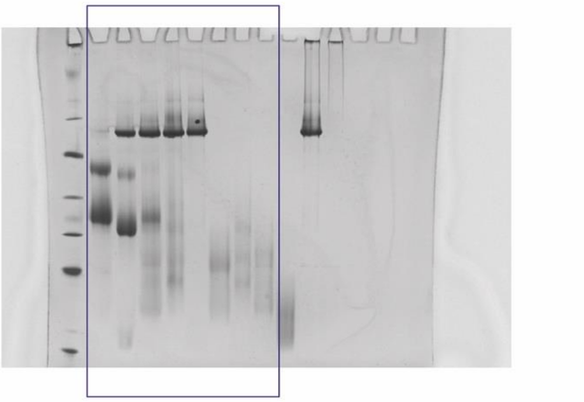

Fig. 5b anti-p97 blot

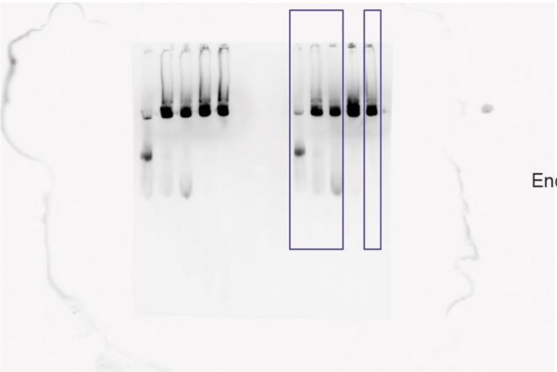

Fig. 4c anti-CD3δ blot

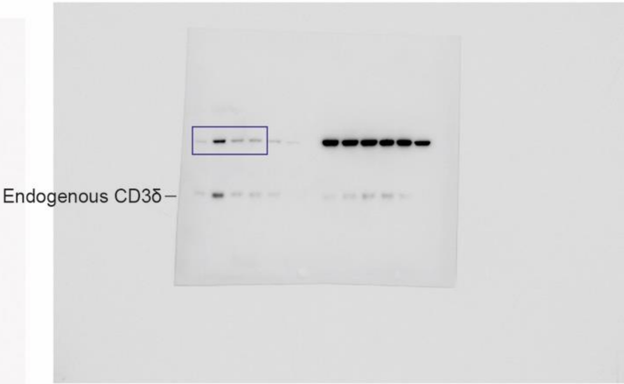

Supplementary Figure 11: Uncropped immunoblots and gels

Uncropped immunblots or Coomassie stained BN-PAGE gels used in the main figures. Blots are labeled according to their appearance in the main figures. Highlighted boxes indicated the cropped images shown in the main figures. Antibodies used to develop the immunoblots are indicated.

Supplementary Table 1. Summary of all PPIs identified by the automated Y2H assay

	Baits	Gene ID	Prey	Gene ID
**1**	p97 1-110 aa	7415	SLC43A3	29015
**2**	p97 1-110 aa	7415	UBE2D1	7321
**3**	p97 111-185 aa	7415	RNF115	27246
**4**	p97 1-208 aa	7415	EPSTI1	94240
**5**	p97 1-208 aa	7415	ELAVL4	1996
**6**	p97 1-208 aa	7415	VAMP2	6844
**7**	p97 1-208 aa	7415	ASPL	79058
**8**	p971-208 aa	7415	ATXN3-Q25	4287
**9**	p97 1-208 aa	7415	ATXN3-Q71	4287
**10**	p97 1-208 aa	7415	NPL4	55666
**11**	p97 1-208 aa	7415	p47	55968
**12**	p97 1-208 aa	7415	UBXD4	165324
**13**	p97 1-208 aa	7415	LZIC	84328
**14**	p97 1-208 aa	7415	UBXD1	80700
**15**	p97 209-460 aa	7415	FAM108C1	58489
**16**	p97 481-766 aa	7415	UBE2D1	7321

aa (amino acids)

Supplementary Table 2. X-ray diffraction, data collection and refinement statistics

	p97-ND1:ASPL-C	p97:ASPL-C_{Δ}
**Data collection**		
Space group	$P2_1$	C222
Cell dimensions		
a, *b*, *c* (Å)	69.8, 132.9, 96.1	143.3, 208.4, 99.1
α, β, γ (°)	90.0, 119.1, 90.0	90.0, 90.0, 90.0
Resolution (Å)*	33.8-2.46 (2.52-2.46)	33.9-3.4 (3.49-3.4)
R_{meas}*	6.4 (59.0)	18.8 (94.5)
< *I* / σ(*I*) >*	16.7 (2.1)	10.8 (2.2)
Completeness (%)*	97.9 (93.2)	99.8 (100)
Redundancy*	2.5 (2.4)	5.0 (5.1)
**Refinement**		
Resolution (Å)	2.46	3.4
No. reflections	55 653	20 785
R_{work} / R_{free}	0.217/0.262	0.225/0.273
No. atoms		
Protein	9 800	7 007
Ligand/ion	73	53
Water	418	19
Mean B factor (Å^2)	44.1	73.0
R.m.s deviations		
Bond lengths (Å)	0.006	0.005
Bond angles (º)	0.988	0.984

*Data in highest resolution shell are indicated in parenthesis.

Supplementary References

1. Huang, D. W., Lempicki, R. a & Sherman, B. T. Systematic and integrative analysis of large gene lists using DAVID bioinformatics resources. *Nat. Protoc.* **4,** 44–57 (2009).
2. DeLaBarre, B. & Brunger, A. T. Complete structure of p97/valosin-containing protein reveals communication between nucleotide domains. *Nat Struct Biol* **10,** 856–863 (2003).
3. Wang, Q. *et al.* Multifunctional roles of the conserved Arg residues in the second region of homology of p97/valosin-containing protein. *J Biol Chem* **280,** 40515–40523 (2005).
